# The Impact of the COVID-19 Pandemic on Physical and Mental Health in China and Spain: Cross-sectional Study

**DOI:** 10.2196/27818

**Published:** 2021-05-21

**Authors:** Cuiyan Wang, María Inmaculada López-Núñez, Riyu Pan, Xiaoyang Wan, Yilin Tan, Linkang Xu, Faith Choo, Roger Ho, Cyrus Ho, Marta E Aparicio García

**Affiliations:** 1 Institute of Cognitive Neuroscience Faculty of Education Huaibei Normal University Huaibei China; 2 Department of Social Work and Differential Psychology Faculty of Psychology Complutense University of Madrid Madrid Spain; 3 Department of Psychological Medicine Yong Loo Lin School of Medicine National University of Singapore Singapore Singapore; 4 Department of Psychological Medicine Institute of Health Innovation and Technology National University of Singapore Singapore Singapore

**Keywords:** anxiety, China, coronavirus, COVID-19, depression, developing countries, knowledge, masks, pandemic, physical, precaution, psychological impact, Spain, stress

## Abstract

**Background:**

Differences in physical and mental health impact across continents during the COVID-19 pandemic are unknown.

**Objective:**

This study compared the levels of impact of COVID-19 on mental health among people from Spain and China and correlated mental health parameters with variables relating to symptoms similar to COVID-19, COVID-19 knowledge, and precautionary measures.

**Methods:**

We collected information on demographic data, physical symptoms, contact history with persons with a confirmed COVID-19 diagnosis, COVID-19 knowledge, and precautionary measures. Participants completed the Impact of Event Scale-Revised (IES-R) and the Depression, Anxiety and Stress Scale–21 Items (DASS-21). To analyze the differences in the mental health parameters, the mean scores between Chinese and Spanish respondents were compared using the independent samples *t* test. The differences in categorical variables between the two samples were analyzed by the chi-square test. Linear regression was used to calculate the univariate associations between the independent variables and mental health parameters for both groups separately, with adjustments made for age, gender, and education.

**Results:**

A total of 1528 participants (Spain: n=687; China: n=841) were recruited. The mean age of the Chinese respondents was 24.73 years (SD 7.60; range 18-65 years), and the mean age of the Spanish respondents was 43.06 years (SD 11.95; range 18-76 years). Spanish participants reported significantly more symptoms similar to COVID-19 infection (eg, fever, sore throat, and breathing difficulties), contact history with COVID-19, higher perceived risk of contracting COVID-19, frequent use of medical services, and less confidence in medical services compared with their Chinese counterparts (*P*<.001). Spanish participants reported significantly higher DASS-21 stress and depression scores, while Chinese participants reported significantly higher IES-R scores (*P*<.001). Chinese participants encountered more discrimination from other countries (*P*<.001). Significantly more Chinese participants reported using face masks than Spanish ones (*P*<.001). More exposure to health information was associated with adverse mental health in Spain (depression: *P*=.02; anxiety: *P*=.02; stress: *P*=.001).

**Conclusions:**

Our study found that Spanish respondents reported higher levels of stress and depression as well as more symptoms and use of medical services. In preparation for the next pandemic, Spain needs to establish a prompt policy to implement rapid response and enhance medical services to safeguard physical and mental health.

## Introduction

### Background

The city of Wuhan was ground zero of the COVID-19 outbreak, which spread to all 23 provinces of China. As a result, China became the first epicenter in Asia during the COVID-19 pandemic [1]. China’s first confirmed COVID-19 case was reported on November 17, 2019. As of May 5, 2020, there were 82,880 confirmed cases, 4633 deaths, and 77,766 recovered cases in China [2]. The Chinese government imposed lockdown measures to restrict travel to prevent transmission. Since the onset of the COVID-19 outbreak, the Chinese government’s response efforts have been swift, and 3 weeks into the epidemic, in an unprecedented move to slow the spread of the virus, a lockdown was imposed in Wuhan on January 23, 2020 [3]. In the Lunar New Year of 2020, the quarantine was extended to additional provinces and cities during the peak travel period. Many Chinese people stayed at home and socially isolated themselves to prevent infection. Due to many new cases in February 2020, the Central Government of China deployed thousands of medical personnel to a rapidly completed hospital specially designed to treat patients with COVID-19 [4]. The Chinese health authority imparted unbiased and clear guidelines on the use of face masks during the early stage of the pandemic [5].

Spain is one of Europe’s COVID-19 epicenters [2]. Spain’s first patient was detected on January 31, 2020, in La Gomera (Canary Islands). However, it was not until February 24 when the virus spread to the peninsula, with cases in Madrid, Catalonia, and the Valencian Community. Since then, the number of COVID-19 infections has progressively increased. There has been a rapid increase in the number of new cases of COVID-19 in Spain since March 2020 [6]. On March 30, 2020, Spain overtook China in terms of the number of infectees [7]. Although there were major differences in government response between Spain and China, no research has been conducted to compare Chinese and Spanish physical and mental health at the beginning of the pandemic. In the early stage of the COVID-19 outbreak, Spanish authorities allowed travel to other European countries without any restrictions as well as mass celebrations [6]. On March 12, 2020, the Spanish government agreed to implement educational and social distancing measures throughout Spain. Two days later, a state of alarm was declared for 14 days to establish measures to protect citizens’ health and safety, contain the progression of the disease, and strengthen the public health system [8]. Among these measures was the mandatory confinement of all citizens. On March 28, the state of alarm was extended until April 12 [9]. On April 9, a third extension was approved until April 25 [10]. On April 18, the government announced a new extension until May 10. On that day, there were a total of 191,726 infections and 20,043 deaths in Spain [11]. More than 10,000 elderly people died in nursing homes, comprising half of Spain’s official death toll [12].

### This Study

The lockdown measures and economic recession associated with the COVID-19 pandemic has put the general public in China and Spain under more pressure than ever before. There has been an atmosphere of anxiety and depression in Spain and China due to the pandemic [13]. Recent studies have reported on depression, anxiety, and stress levels among Chinese [14-17], Thai [18], and Vietnamese [19] populations during the pandemic in Asia. A recent study found that the impact of anxiety on adopting social distancing did not vary between people in the United Kingdom and Hong Kong [20]. The psychological impact of other preventive measures between people in Asia and Europe require further study. As a result, we proposed a study to conduct an in-depth analysis of physical and mental health in China and Spain during the pandemic. The theoretical framework of this study was based on the chain mediation model, which shows the need for health information, and the perceived impacts of the pandemic were sequential mediators between physical symptoms resembling COVID-19 infection and consequent mental health status [21].

We aimed (1) to compare levels of physical health (eg, COVID-19–related symptoms) and mental health status (eg, depression, anxiety, stress, and psychological impact) between Spanish and Chinese survey respondents during the pandemic; and (2) to correlate psychological impact, depression, anxiety, and stress scores with variables relating to physical symptoms, COVID-19 knowledge and views (eg, knowledge of COVID-19 transmission, confidence in medical services, etc), prevention measures (eg, face mask use, hand hygiene), and information needs (eg, symptoms, prevention methods, the effectiveness of drugs and vaccines) among Spanish and Chinese respondents. The null hypothesis was that there would be no difference in survey scores between Spanish and Chinese participants. The other null hypothesis was that physical symptoms resembling COVID-19, higher confidence in medical services, perceived higher chances of survival, satisfaction with health information, and adoption of precautionary measures had no significant association with lower survey scores in both countries.

## Methods

### Study Participants

This cross-cultural study compared the mental and physical health status and the psychological impact between Spanish and Chinese people. The recruitment period was from February 28 to March 1, 2020, in China, a month after the Chinese government declared a lockdown of Wuhan. For Spain, data collection was from April 14 to 18, 2020, a month after the Spanish government declared the state of alarm. A snowball sampling strategy was utilized to recruit participants from the general public in Spain and China. Recruitment started with a set of initial respondents who were associated with the Huaibei Normal University of China and the Complutense University of Madrid, who referred other participants by email and social networks including colleagues, relatives, classmates, and friends. These individuals, in turn, referred other participants across different cities in China and Spain. Inclusion criteria restricted participation to respondents who could provide online written consent, had access to the internet, and were above 18 years of age. Exclusion criteria included respondents who were illiterate or had no access to the internet.

### Study Procedure

Potential participants were electronically invited by respondents who had completed the questionnaires. The respondents completed the online survey via SurveyStar in China and Google Forms (Google LLC) in Spain.

### Study Outcomes

The variables used in this study have been used previously in research on the severe acute respiratory syndrome (SARS); thus, the content of variables have been validated [22]. This study used a questionnaire with psychometric properties established at the beginning and peak of the COVID-19 epidemic [3,15]. The COVID-19 questionnaire had 43 items and covered the following areas: (1) demographic information, (2) symptoms similar to COVID-19 infection in the past 2 weeks, (3) contact history with COVID-19 in the past 14 days, (4) knowledge about COVID-19, and (5) preventive measures against COVID-19 in the past 2 weeks. Responses depended on the questions (eg, yes or no, Likert scale based on frequency). To ensure high data quality, the structured questionnaire was developed in English in such a way that it included all relevant variables to meet the objective; to ensure consistency in the questionnaire, it was first prepared in English and then translated into Chinese and Spanish by fluent speakers of both languages and then translated back to English. Data were checked for completeness and consistency before processing and analysis.

Demographic data on age, gender, education, household size, marital status, parental status, and residential city in the past 2 weeks were collected. Physical symptoms similar to COVID-19 infection included cough, fever, gastrointestinal symptoms, and other symptoms. Respondents also stated their past medical history, health service use, quarantine by health authority, and recent testing for COVID-19. Knowledge and views related to COVID-19 included confidence in medical services, health information satisfaction, the practice of hand hygiene and face mask use, time spent viewing health information, transmission route, and the likelihood of contracting and surviving COVID-19.

The Chinese and Spanish versions of the Impact of Event Scale-Revised (IES-R) and the Depression, Anxiety and Stress Scale–21 Items (DASS-21) were used to assess mental health. The IES-R has 22 items, and it was previously validated in the European and Asian populations to assess impact when exposed to the COVID-19 pandemic [23-25]. The IES-R measures avoidance, intrusion, and hyperarousal [26]. The IES-R items used the following Likert-scales responses: 0=not at all, 1=a little bit, 2=moderately, 3=quite a bit, and 4=extremely. A total IES-R score of 0 to 23 is considered normal, 24 to 32 is mild, 33 to 36 is moderate, and ≥37 is severe [27]. A total IES-R score of >24 is the cut-off score for posttraumatic stress disorder (PTSD) symptoms [28]. In this study, the Cronbach alphas of IES-R for the Chinese and Spanish surveys were .949 and .948, respectively.

DASS-21 scores were calculated based on a previous Asian study [29]. DASS-21 has 21 items, and it has been used to assess mental health in Chinese [30,31] and Spanish [32] populations. The responses were indicated using the following Likert scale: 0=did not apply to me at all, 1=applied to me to some degree or some of the time, 2=applied to me to a considerable degree or a good part of the time, and 3=applied to me very much or most of the time. In this study, the Cronbach alpha for the Chinese version of DASS-21 was as follows: stress: .888, anxiety: .845, and depression: .878; for the Spanish version, the Cronbach alpha was as follows: stress: .895, anxiety: .876, and depression: .89. DASS-21 and IES-R have been previously used in COVID-19 research [3,17,24,33].

### Statistical Analysis

Statistical analysis was performed on SPSS Statistics 21.0 (IBM Corp). Descriptive statistics were reported for demographics, COVID-19 symptoms, health service utilization, knowledge about COVID-19, and preventive measures. To analyze the differences in the mental health parameters, the differences in mean scores between the Chinese and Spanish respondents were compared by the independent samples *t* test. The differences in categorical variables between the two samples were analyzed by the chi-square test. Linear regression was used to calculate the univariate associations between independent variables and mental health parameters for the Chinese and Spanish respondents separately with adjustment for age, gender, and education. The significance level was set at *P*<.05.

### Ethics Statement

The institutional review board of the Complutense University of Madrid (Pr_2019_20_027) and the Huaibei Normal University (HBU-IRB-2020-002) approved the studies for Spain and China, respectively. All respondents signed the informed consent electronically. The collected information was anonymous and treated as confidential.

## Results

### Comparison of Mental Health Status Between Spanish and Chinese Participants

In Spain, 687 respondents completed the questionnaires (completion rate: 94.0%). We excluded 4 incomplete questionnaires in the Chinese sample, which left a total of 841 out of 845 (99.5%) valid questionnaires. Respondents from China came from 159 cities, and respondents from Spain came from all 19 autonomous regions. As a result, the total number of respondents from both countries was 1528. In terms of age, 338 respondents from China were aged 18-21 years, 400 respondents were 22-30 years, 37 respondents were 31-40 years, 51 respondents were 41-49 years, and 15 respondents were ≥50 years. In Spain, 27 respondents were aged 18-21 years, 91 respondents were 22-30 years, 158 respondents were 31-40 years, 200 respondents were 41-49 years, and 211 respondents were ≥50 years.

Table 1 compares the mental health status of the Spanish and Chinese samples. For the DASS-21 stress subscale (China: mean 7.79, SD 7.90; Spain: mean 14.23, SD 10.04), Spanish respondents reported significantly higher scores than Chinese respondents (*t*_1287.27_=–13.70, *P*<.001, 95% CI –7.36 to –5.52). For the DASS-21 depression subscale (China: mean 6.28, SD 7.30; Spain: mean 8.61, SD 8.80), Spanish respondents had significantly higher depression scores (*t*_1330.70_=–5.55, *P*<.001, 95% CI –3.15 to –1.51). For the DASS-21 anxiety subscale (China: mean 6.07, SD 6.87; Spain: mean 6.78, SD 8.23), there was no significant difference between the two countries (*t*_1335.75_=–1.79, *P*=.07, 95% CI –1.48 to 0.07). For the IES-R scale (China: mean 30.69, SD 16.21; Spain: mean 27.62, SD 18.67), Spanish participants had significantly lower scores than their Chinese counterparts (*t*_1367.38_=3.39, *P*=.001, 95% CI 1.30-4.85). Nevertheless, the mean IES-R scores of respondents from both countries were greater than 24 points (ie, the cut-off score for PTSD symptoms). Table S1 in Multimedia Appendix 1 compares the mental health status of both samples across different age groups. Both Chinese and Spanish respondents showed significant differences in IES-R scores based on age category, with the 18-21 years age group having significantly higher IES-R scores than the 41-49 years and ≥50 years age groups (Spain: 18-21 years, *P*=.001; 41-49 years, *P*=.02; China: 18-21 years, *P*=.02; 41-49 years, *P*=.49). For the DASS-21 scores, Spanish respondents showed significant differences, with the 18-21 years age group reporting significantly higher depression (*P*=.002), anxiety (*P*=.02), and stress (*P*<.001) scores than those ≥50 years (Table S1, Multimedia Appendix 1).

**Table 1:** Scores of the Depression, Anxiety and Stress Scale–21 Items (DASS-21) and Impact of Event Scale-Revised (IES-R) for Spanish and Chinese respondents.

Country	DASS-21				IES-R, score (SD)
	Depression, score (SD)	Anxiety, score (SD)	Stress, score (SD)		
China	6.28 (7.30)	6.07 (6.87)	7.79 (7.90)	30.69 (16.21)	
Spain	8.61 (8.80)	6.78 (8.23)	14.23 (10.04)	27.62 (18.67)	

### Association Between Variables

#### Demographic Characteristics and Mental Health Status

More than half the Spanish respondents were women (n=541, 78.7%), had a family size of 3 to 5 people (n=368, 53.6%), were well educated (n=516, 75.8% with a bachelor or higher degree), and were married (n=74, 10.8%). Similarly, more than half the Chinese respondents were women (n=631, 75%), had a family size of 3 to 5 people (n=676, 80.4%), were well educated (n=754, 89.2% with a bachelor or higher degree), and were married (n=699, 83.5%). A significantly higher proportion of Chinese respondents were younger, lived with more than 6 people in the same household, and had a child over 16 years (*P*<.001) (Table S2, Multimedia Appendix 1).

Among the Spanish respondents, the male gender was a protective factor associated with a lower score for the DASS-21 stress (*P*=.004) and depression (*P*=.02) subscales and IES-R (*P*=.001) while age <50 years and having a child over 16 years of age were risk factors associated with a higher score for IES-R (*P*=.02)and some of the DASS-21 subscales (stress: *P*<.001; anxiety: *P*=.03) (Table S1, Multimedia Appendix 2). Among the Chinese respondents, the male gender was associated with a lower IES-R score (*P*=.01) but a higher DASS-21 depression score (*P*=.002). Chinese people who lived in a household with 3 to 5 people (*P*=.04) and more than 6 people (*P*=.03) were associated with a higher score of IES-R compared to those who lived alone. This association was not observed in the Spanish sample.

#### Physical and Mental Health Parameters

Previous studies have established the association between physical symptoms and psychological outcomes during the COVID-19 outbreak [24]. For symptoms resembling COVID-19, significantly more Spanish individuals reported fever (*P*<.001), chills (*P*<.001), headache (*P*<.001), myalgia (*P*<.001), cough (*P*<.001), breathing difficulty (*P*<.001), dizziness (*P*<.001), coryza (*P*<.001), sore throat (*P*<.001), nausea and vomiting (*P*<.001), recent consultation with a doctor (*P*<.001), recent hospitalization and quarantine (IES-R: *P*=.03; anxiety: *P*=.01), recent COVID-19 testing (*P*<.001), chronic illness (*P*<.001), direct (*P*<.001) and indirect (*P*<.001) contact with someone who was COVID-19 positive, and direct contact with contaminated materials (*P*<.001) compared to the Chinese sample (Table S3, Multimedia Appendix 1). Nevertheless, significantly more Spanish respondents rated good physical health compared with the Chinese sample (*P*<.001).

Linear regression showed that headache, myalgia, and dizziness were associated with higher DASS-21 stress, anxiety, and depression and IES-R scores in both countries (Spain—headache: IES-R, *P*=.002; stress, *P*=.001; anxiety, *P*<.001; depression, *P*=.001; myalgia: IES-R, *P*<.001; stress, *P*<.001; anxiety, *P*=.002; depression, *P*=.02; dizziness: IES-R, *P*=.03; stress, *P*=.05; anxiety, *P*=.003; depression, *P*=.02; China—headache: IES-R, *P*=.02; stress, *P*<.001; anxiety, *P*<.001; depression, *P*=.001; myalgia: IES-R, *P*=.007; stress, *P*<.001; anxiety, *P*<.001; depression, *P*<.001; dizziness: IES-R, *P*<.001; stress, *P*<.001; anxiety, *P*<.001; depression, *P*=.007) (Table S2, Multimedia Appendix 2). In contrast, myalgia, sore throat, and gastrointestinal symptoms were associated with higher DASS-21 stress, anxiety, and depression scores in both countries (Spain—myalgia: stress, *P*<.001; anxiety, *P*=.002; depression, *P*=.02; sore throat: stress, *P*=.01; anxiety, *P*<.001; depression, *P*=.03; gastrointestinal symptoms: stress, *P*=.004; anxiety, *P*≤.001; depression, *P*=.007; China—myalgia: stress, *P*<.001; anxiety, *P*<.001; depression, *P*<.001; sore throat: stress, *P*<.001; anxiety, *P*<.001; depression, *P*<.001; gastrointestinal symptoms: stress, *P*<.001; anxiety, *P*=.002; depression, *P*<.001). Recent consultation with a doctor and quarantine were associated with greater psychological impact and anxiety (consultation with a doctor; IES-R, *P*=.02; stress, *P*=.002; quarantine: IES-R, *P*=.03; anxiety, *P*=.01) in Spanish respondents only.

#### Knowledge and Views of COVID-19 and Mental Health Parameters

Spanish and Chinese respondents held significantly different views regarding knowledge and views related to COVID-19 (Table S4, Multimedia Appendix 1). For the routes of transmission, there were significantly more Spanish respondents who agreed that droplets transmitted the coronavirus (*P*=.01), as well as contact via contaminated objects (*P*<.001), but significantly more Chinese individuals agreed with airborne transmission (*P*<.001). In terms of detection and risk of contracting COVID-19, there were significantly more Spanish people who were not confident about the competency of doctors to diagnose COVID-19 (*P*<.001) and perceived a greater risk of contracting COVID-19 (*P*<.001) as well as greater chances of survival after COVID-19 infection (*P*<.001). More Spanish respondents were concerned about their relatives contracting COVID-19 (*P*<.001). There were significantly more Spanish participants who were unsatisfied with the amount of health information they received (*P*<.001). There were significantly more Chinese respondents who felt discriminated against by other countries (*P*<.001).

Spanish and Chinese respondents showed different results in the linear regression analysis (Table S3, Multimedia Appendix 2). In terms of the level of confidence in doctors to diagnose COVID-19, Chinese individuals who were somewhat confident and very confident were associated with lower scores on the DASS-21 depression subscale (very confident: *P*=.002; somewhat confident: *P*=.01). Chinese individuals who perceived a lower chance of contracting COVID-19 were associated with a lower DASS-21 depression score (*P*=.04), and those who perceived a very high likelihood of survival were associated with lower DASS-21 depression and IES-R scores (depression: *P*=.02; IES-R: *P*=.04). Chinese individuals who were satisfied with the health information they received were significantly associated with lower DASS-21 depression, anxiety, and stress scores (depression: very confident, *P*<.001; somewhat confident, *P*=.002; anxiety: very confident, *P*=.003; somewhat confident, *P*=.01: stress: very confident, *P*<.001; somewhat confident, *P*=.001). The above significant association was not found among Spanish respondents (confidence in doctors—depression: very confident, *P*=.21, somewhat confident, *P*=.44; chance of contracting COVID-19: *P*=.14; likelihood of survival after contracting COVID-19: depression, *P*=.32; IES-R, *P*=.96; satisfaction with health information—depression: very satisfied, *P*=.67; somewhat satisfied, *P*=.42; anxiety: very satisfied, *P*=.92; somewhat satisfied, *P*=.66; stress: very satisfied, *P*=.65; somewhat satisfied, *P*=.45). In contrast, Spanish respondents who spent less than 1 hour per day monitoring COVID-19 information were significantly associated with lower DASS-21 depression, anxiety, and stress scores and IES-R scores (depression: *P*=.01; anxiety: *P*=.001; stress: *P*=.02; IES-R: *P*=.02). Spanish individuals who felt discriminated were associated with higher DASS-21 depression, anxiety, and stress scores and IES-R scores (depression: *P*=.01; anxiety: *P*=.003; stress: *P*<.001; IES-R: *P*<.001).

#### Precautionary Measures and Mental Health Parameters

Spanish and Chinese respondents demonstrated significantly different precautionary measures (Table S5, Multimedia Appendix 1). There were significantly more Spanish individuals who reported covering their mouths when coughing and sneezing (*P*<.001), avoiding the sharing of utensils (*P*=.01), and practicing hand hygiene (*P*<.001). More Spanish respondents felt that the COVID-19 pandemic caused unnecessary worry (*P*<.001). In contrast, there were significantly more Chinese respondents who washed their hands immediately after coughing or sneezing (*P*<.001), agreed to wear a face mask (*P*<.001), and practiced hand hygiene after touching contaminated objects (*P*<.001).

The linear regression analysis found that the avoidance of sharing utensils, covering one’s mouth when coughing and sneezing, practicing hand hygiene, and using a face mask were significantly associated with lower DASS-21 and IES-R scores (avoidance of sharing utensils: depression, *P*=.01; stress, *P*=.02; IES-R, *P*=.01; covering mouth when sneezing and coughing: depression, *P*<.001; anxiety, *P*=.01; stress, *P*=.01; hand hygiene—wash hands with soap: depression, *P*<.001; anxiety, *P*=.002; stress, *P*=.001; wash hands immediately after coughing and sneezing: depression, *P*<.001; anxiety, *P*<.001; stress, *P*<.001; IES-R, *P*=.01; wash hands immediately after touching contaminated objects: depression, *P*=.001; anxiety, *P*=.01; stress, *P*=.002; wearing a face mask: depression, *P*<.001; anxiety, *P*=.02; stress, *P*<.001) among Chinese respondents (Table S4, Multimedia Appendix 2). Among Spanish respondents, covering one’s mouth when coughing and sneezing and hand hygiene measures were associated with lower DASS-21 anxiety and stress subscale scores, respectively (covering mouth when coughing and sneezing: anxiety, *P*=.02; stress, *P*=.02; hand hygiene—wash hand with soap: anxiety, *P*=.04; stress, *P*=.01). Wearing a face mask was also associated with lower IES-R and DASS-21 stress scores in Spanish respondents (IES-R: *P*=.01; stress: *P*=.047). Spanish respondents who felt the COVID-19 pandemic caused unnecessary worry were associated with higher DASS-21 depression, anxiety, and stress scores (depression: *P*=.01; anxiety: *P*=.03; stress: *P*=.04). In contrast, Chinese respondents who felt that the COVID-19 pandemic had caused too much unnecessary worry were associated with lower IES-R scores (*P*=.01).

#### Health Information About COVID-19 and Mental Health Parameters

Spanish and Chinese participants were significantly different in terms of information needs pertaining to COVID-19. There were significantly more Chinese participants who needed regular updates; more personalized information; and data on symptoms related to COVID-19, prevention methods, management and treatment methods, the effectiveness of drugs and vaccines, the number of infected by geographical locations, travel advice, and transmission methods compared to Spanish participants (*P*<.001) (Table S6, Multimedia Appendix 1). In contrast, there were significantly more Spanish respondents who needed information on other countries’ strategies and responses than Chinese respondents (*P*<.001).

Health information on prevention methods, as well as more personalized information on transmission methods, information related to COVID-19 in general, and the effectiveness of drugs and vaccines, were associated with higher IES-R scores or higher scores for one of the DASS-21 subscales among Spanish respondents only (prevention methods: IES-R, *P*<.001; stress, *P*<.001; anxiety, *P*<.001; mode of transmission: IES-R, *P*<.001; stress, *P*<.001: anxiety, *P*<.001; depression, *P*=.0.01; personalized information: IES-R, *P*<.001; stress, *P*=.003; anxiety, *P*=.001; depression, *P*=.004; effectiveness of medication: IES-R, *P*=.003) (Table S5, Multimedia Appendix 2). Information on management methods and transmission methods was associated with higher IES-R scores in both Spanish and Chinese respondents (Spain: management methods, *P*<.001; transmission methods, *P*<.001; China: management methods, *P*=.04; transmission methods, *P*=.01). Travel advice and information on other countries’ responses were associated with lower depression scores only in China (travel advice: *P*=.02; information on other countries’ responses: *P*=.03).

## Discussion

### Principal Findings

This is the first study to compare the physical and mental health of citizens from two COVID-19 epicenters in Europe and Asia. Spain and China faced different problems. In Spain, the number of COVID-19 cases and deaths was 5494 (98 times that of China) and 558 (186 times that of China) per 1 million population, respectively, in May 2020 [2]. The research findings rejected the null hypothesis. A significantly higher proportion of Spanish respondents reported symptoms similar to COVID-19 infection, contact history with COVID-19, and higher perceived risk of contracting COVID-19. More Spanish respondents also utilized medical services recently, but they reported less confidence in their medical services. Spanish respondents were more dissatisfied with health information; the time and types of health information were associated with adverse mental health. Spanish respondents reported significantly higher levels of depression and stress during the pandemic than Chinese participants, especially younger Spanish people (18-40 years).

Protective factors against adverse mental health in Spain included the male gender, living with an older child, and spending less time monitoring COVID-19 information. In China, the number of COVID-19 cases and deaths were 58 and 3 per 1 million population, respectively, in May 2020 [2]. Although China has a relatively lower number of COVID-19 cases and death per capita, significantly more Chinese respondents reported a higher psychological impact and discrimination by other countries. Protective factors against adverse mental health in China include smaller household sizes, higher confidence levels in their doctors, wearing face masks, and obtaining information on travel advice and other countries’ responses during the pandemic. For both countries, risk factors for adverse mental health include the presence of symptoms similar to COVID-19 infection (eg, headache, myalgia, sore throat, gastrointestinal symptoms). Protective factors for both countries included certain precautionary behaviors (eg, covering one’s mouth when coughing or sneezing, certain hand hygiene measures).

This study has provided an understanding of the differences between China and Spain, which will allow us to better prepare for the next pandemic. The higher levels of adverse mental health and lower confidence in Spanish medical services were due to the rising number of infected health care workers in Spain and the lack of effective coordination between different hospitals [34]. In contrast, the Chinese government rapidly deployed medical personnel and treated patients with COVID-19 at rapidly built hospitals [4]. This prompt action had restored public confidence in the health care system. Spain and other European countries shared four common characteristics in the early stage of the pandemic: (1) the lack of personal protection equipment (PPE) for health care workers, (2) the delay in response strategy, (3) an overstretched health care system with a shortage of hospital beds, and (4) the failure to protect vulnerable nursing home residents from COVID-19 [35]. Following the COVID-19 pandemic, European countries need to rectify the insufficiencies in the number of available health care professionals, hospital beds, and medical equipment (eg, PPE, mechanical ventilators) caused by long-term underinvestment in health services following the 2008 financial crisis [36].

Many research studies supported the benefits of face masks in blocking virus transmission in aerosols but were opposed by erroneous judgment [37]. Wearing a face mask can impede the spread of the virus from asymptomatic patients with COVID-19 [38]. Nevertheless, this study found that Spanish respondents were significantly less likely to wear face masks than Chinese ones. The Chinese government provided health information on COVID-19 transmission via contact, droplets, and through the air; personal preventive measures (eg, wearing a face mask, hand hygiene, other personal precaution); and public health measures (eg, good ventilation, social distancing, COVID-19 testing for the general population) [16]. Furthermore, the Chinese government emphasized the benefits of mask wearing due to airborne transmission and made such practice mandatory in February 2020 to reduce the coronavirus spread [39]. Besides potential benefits on physical health, wearing face masks could offer psychological benefits associated with lower IES-R and DASS-21 stress scores among Chinese and Spanish respondents. A recent epidemiological study compared the incidence of COVID-19 per 1 million in Hong Kong with a high prevalence of community-wide masking with non–mask-wearing countries. This study found that the incidence of COVID-19 in Hong Kong was 129.0 per 1 million population, which was much lower than the incidence rate of Spain (2983.2 per million population, 23 times the incidence rate of Hong Kong) [40]. During the beginning stage of the COVID-19 pandemic, Europeans held ambivalent views toward face masks due to cultural reasons. It was generally difficult for Europeans to accept the need for mask use by healthy people since mask wearing suggests vulnerability to sickness and concealment of identity. Due to the collective culture of China, Chinese individuals are more aware of the importance of wearing masks and the responsibility associated with the need to protect their health and the health of others—this sentiment is more pronounced in China than it is in Europe. Our findings suggest that there is a need for health education with scientific information from Spanish health authorities on the use and benefits of face masks during the pandemic and implement measures to reduce social stigma.

Chinese participants reported a significantly higher level of the psychological impact associated with the COVID-19 pandemic. China, the epicenter in Asia in the early stage of the pandemic, faced other unique problems not shared by its European counterparts. A significantly higher number of Chinese people felt they were discriminated against by other countries due to COVID-19 through negative media comments. The editor-in-chief of *The* *Lancet*, Richard Horton, expressed concern for the discrimination faced by China, saying that while it is important to understand the origin and interspecies transmission of the coronavirus, it is both unhelpful and unscientific to attribute China as the origin of the COVID-19 pandemic and seek a patient zero, as such efforts could be highly stigmatizing and discriminatory [41]. Global cooperation involving an exchange of expertise, adopting effective prevention strategies, and sharing resources and technologies across different countries to form a united front to tackle the COVID-19 pandemic remains a work in progress. As physical symptoms resembling COVID-19 infection (eg, headache, myalgia, sore throat, gastrointestinal symptoms) were associated with adverse mental health in both epicenters, the lack of testing for the coronavirus could worsen anxiety and stress. There is an urgent need to develop accurate, rapid diagnostic tests for use in general practitioners’ clinics and community settings. Nevertheless, there is a need for a culturally sensitive approach to improve mental health during the pandemic. Further research is required to understand the perception of Spanish individuals toward health information related to COVID-19 and explore the underlying reasons between adverse mental health and health information (eg, symptoms, prevention methods, transmission methods, and personalized information). Furthermore, online psychological intervention such as internet-delivered cognitive behavioral therapy is a cost-effective treatment [42] and can reduce psychological symptoms [43] and challenge negative thoughts. Online psychological treatment can be conducted without face-to-face contact, thereby adhering to lockdown and social distancing measures during the pandemic.

### Strengths and Limitations

The main strength of this study lies in the fact that we performed in-depth analyses and studied the relationship between psychological outcomes and other variables related to COVID-19 in the two largest epicenters in Europe and Asia during the early stage of the pandemic. However, there are several limitations to be considered when interpreting the results. One major limitation was the potential risk of sampling bias, although respondents came from 159 cities from China and all 19 autonomous regions in Spain. This bias could be due to the online administration of the questionnaires; further, the majority of respondents from both countries had a good educational background and internet access. We could not reach out to potential respondents without internet access. The research findings should be interpreted with caution as the respondents might not be representative of the population, and the demographic data were not normally distributed. Nevertheless, it is important to compare mental health between two epicenters across two continents during the early stage of the pandemic as such a study cannot be done after the pandemic. Another major limitation is that Spanish and Chinese respondents were not matched by age as the Spanish respondents were significantly older and more likely to be unmarried compared to their Chinese counterparts. The differences observed between the Spanish and Chinese participants could be due to intrinsic differences in age and other demographic factors. Another limitation is that we were unable to calculate the response rate. For potential respondents who were not keen to participate in the online survey, no response was recorded, and we could not collect any information from them due to ethical requirements. Furthermore, we could only include one country from Asia and Europe because not all universities had agreements for collaboration and research data exchange. Our findings cannot be generalized to other epicenters in Asia and Europe (eg, Italy and the Lombardy region) [44]. Finally, this is a cross-sectional study, and we demonstrated an association but not a causal relationship between the independent variables and psychological outcomes.

### Conclusions

COVID-19 epicenters in Europe and Asia faced different challenges. Due to a higher number of COVID-19 cases per capita in Spain and lack of previous health care system experience in dealing with the SARS outbreak, Spanish respondents reported more physical symptoms, contact history with COVID-19, higher perceived risk of contracting COVID-19, and frequent use of but less confidence in medical services. Spanish participants reported higher levels of stress and depression, while Chinese participants reported higher levels of psychological impact. Chinese respondents encountered more discrimination from other countries. There were cultural differences regarding the use of face masks and satisfaction with health information related to COVID-19. Spanish respondents were less likely to wear face masks. Spanish respondents were more dissatisfied with health information; the timing and types of health information were associated with adverse mental health parameters. Our findings also confirmed that physical symptoms resembling COVID-19 infection (eg, headache, myalgia, sore throat, gastrointestinal symptoms) were associated with adverse mental health in China and Spain.

## Appendix

Multimedia Appendix 1 Supplementary tables (part 1).

Multimedia Appendix 2 Supplementary tables (part 2).

## Abbreviations

DASS-21: Depression, Anxiety and Stress Scale–21 Items
IES-R: Impact of Event Scale-Revised
PTSD: posttraumatic stress disorder
SARS: severe acute respiratory syndrome
