# Supplementary material for: The Impact of the COVID-19 Pandemic on Physical and Mental Health in China and Spain: Cross-sectional Study
Source: JMIR Form Res. 2021 May 21;5(5):e27818. doi: 10.2196/27818 (PMC8143872; doi:10.2196/27818)
Supplement: Multimedia Appendix 1 [file formative_v5i5e27818_app1.docx]

| **Table S1A Comparison of IES-R and DASS-21 scores across age groups among Chinese respondents** | | | | | | | | | | | | | | | | | | | | | | | |
| --- | --- | --- | --- | --- | --- | --- | --- | --- | --- | --- | --- | --- | --- | --- | --- | --- | --- | --- | --- | --- | --- | --- | --- |
| **Age groups** | IES-R | | | | |  | DASS-21 Stress | | | | |  | DASS-21 Anxiety | | | | |  | DASS-21 Depression | | | | |
|  | *n* | *M±SD* | *F* | *p* | *LSD* |  | *n* | *M±SD* | *F* | *p* | *LSD* |  | *n* | *M±SD* | *F* | *p* | *LSD* |  | *n* | *M±SD* | *F* | *p* | *LSD* |
| 1. [18-21] | 338 | 2.72±1.26 | 3.250 | 0.012 | 1>2  1>4  1>5 |  | 338 | 1.42±0.72 | 0.749 | 0.559 | - |  | 338 | 1.72±1.14 | 1.563 | 0.182 | - |  | 338 | 1.48±0.90 | 1.562 | 0.182 | - |
| 1. [22-30] | 400 | 2.52±1.29 |  |  |  |  | 400 | 1.48±0.76 |  |  |  |  | 400 | 1.83±1.23 |  |  |  |  | 400 | 1.63±1.03 |  |  |  |
| 1. [31-40] | 37 | 2.57±1.37 |  |  |  |  | 37 | 1.43±0.87 |  |  |  |  | 37 | 1.76±1.23 |  |  |  |  | 37 | 1.57±0.96 |  |  |  |
| 1. [41-49] | 51 | 2.20±1.30 |  |  |  |  | 51 | 1.31±0.71 |  |  |  |  | 51 | 1.45±0.88 |  |  |  |  | 51 | 1.35±0.82 |  |  |  |
| 1. ≥50 | 15 | 1.93±1.22 |  |  |  |  | 15 | 1.47±0.64 |  |  |  |  | 15 | 1.47±1.13 |  |  |  |  | 15 | 1.53±0.83 |  |  |  |

| **Table S1B Comparison of IES-R and DASS-21 scores across age groups among Spanish** | | | | | | | | | | | | | | | | | | | | | | | |
| --- | --- | --- | --- | --- | --- | --- | --- | --- | --- | --- | --- | --- | --- | --- | --- | --- | --- | --- | --- | --- | --- | --- | --- |
| **Age groups** | IES-R | | | | |  | DASS-21 Stress | | | | |  | DASS-21 Anxiety | | | | |  | DASS-21 Depression | | | | |
|  | *n* | *M±SD* | *F* | *p* | *LSD* |  | *n* | *M±SD* | *F* | *p* | *LSD* |  | *n* | *M±SD* | *F* | *p* | *LSD* |  | *n* | *M±SD* | *F* | *p* | *LSD* |
| 1. [18-21] | 27 | 2.81±1.24 | 4.292 | 0.002 | 1>4>5  2>5  3>5 |  | 27 | 2.52±1.12 | 10.743 | <0.001 | 1>4>5  2>5  3>4  3>5 |  | 27 | 2.33±1.49 | 3.622 | 0.006 | 1>5  3>5  4>5 |  | 27 | 2.30±1.35 | 4.337 | 0.002 | 1>5  3>5  1>4  2>5 |
| 2. [22-30] | 91 | 2.35±1.36 |  |  |  |  | 91 | 2.30±1.18 |  |  |  |  | 91 | 1.87±1.37 |  |  |  |  | 91 | 1.97±1.41 |  |  |  |
| 3. [31-40] | 158 | 2.38±1.33 |  |  |  |  | 158 | 2.29±1.23 |  |  |  |  | 158 | 2.15±1.54 |  |  |  |  | 158 | 1.91±1.22 |  |  |  |
| 4. [41-49] | 200 | 2.26±1.32 |  |  |  |  | 200 | 2.03±1.07 |  |  |  |  | 200 | 1.96±1.37 |  |  |  |  | 200 | 1.73±1.19 |  |  |  |
| 5. ≥50 | 211 | 1.96±1.27 |  |  |  |  | 211 | 1.67±0.93 |  |  |  |  | 211 | 1.66±1.23 |  |  |  |  | 211 | 1.55±1.00 |  |  |  |

LSD = Least Significant Difference

LSD = Least Significant Difference

| **Table S2: Comparison of demographic characteristics between Spanish and Chinese respondents (N= 1528)** | | | | | |
| --- | --- | --- | --- | --- | --- |
| **Demographic Characteristics** | Spain  (N=687) |  | China  (N=841) | Chi-square (χ^2^) | *p*-value |
|  | N(%) |  | N(%) |  |  |
| **Gender** |  |  |  |  |  |
| Male | 146(21.3) |  | 210(25.0) | 3.515 | *p*=0.061 |
| Female | 541(78.7) |  | 631(75.0) |  |  |
| **Age range** |  |  |  |  |  |
| [18-21] | 27(3.9) |  | 338(40.2) | 791.585 | *p*<0.001*** |
| [22-30] | 91(13.2) |  | 400(47.5) |  |  |
| [31-40] | 158(23.1) |  | 37(4.4) |  |  |
| [41-49] | 200(29.1) |  | 51(6.1) |  |  |
| ≥50 | 211(30.7) |  | 15(1.8) |  |  |
| **Status as a parent** |  |  |  |  |  |
| Has a child 16 years or below | 323(47.0) |  | 142(16.9) | 169.003 | *p*<0.001*** |
| Has a child older than 16 years | 212(30.9) |  | 433(51.5) |  |  |
| No children | 152(22.1) |  | 266(31.6) |  |  |
| **Household size** |  |  |  |  |  |
| 6 people or more | 7(1.0) |  | 120(14.3) | 384.745 | *p*<0.001*** |
| 3-5 people | 368(53.6) |  | 676(80.4) |  |  |
| 2 people | 205(29.8) |  | 41(4.8) |  |  |
| 1 person | 107(15.6) |  | 4(0.5) |  |  |
| **Educational level** |  |  |  |  |  |
| Primary school or below | 13(1.9) |  | 53(6.3) | NA | NA |
| Secondary school | 153(22.3) |  | 38(4.5) |  |  |
| University – Bachelor | 264(38.4) |  | 632(75.1) |  |  |
| University – Master or Doctorate | 257(37.4) |  | 118(14.1) |  |  |
| **Marital status** |  |  |  |  |  |
| Single | 304(44.4) |  | 134(15.9) | NA | NA |
| Married | 74(10.8) |  | 699(83.1) |  |  |
| Divorced/Separated | 298(43.5) |  | 5(0.6) |  |  |
| Widowed | 9(1.3) |  | 3(0.4) |  |  |

* *p*<0.05, ***p*<0.01, ****p*<0.001

| **Table S3. Comparison of physical symptoms related to COVID-19, health status and contact history between Spanish and Chinese respondents (N= 1528)** | | | | | |
| --- | --- | --- | --- | --- | --- |
| **Physical symptoms and health status** | Spain  (N=687) |  | China  (N=841) | Chi-square (*χ^2^*) | *p*-value |
|  | N(%) |  | N(%) |  |  |
| **Persistent fever** |  |  |  |  |  |
| Yes | 14(2.0) |  | 1(0.1) | 14.324 | *p*<0.001*** |
| No | 673(98.0) |  | 840(99.9) |  |  |
| **Chills** |  |  |  |  |  |
| Yes | 56(8.2) |  | 11(1.3) | 42.237 | *p*<0.001*** |
| No | 631(91.8) |  | 830(98.7) |  |  |
| **Headache** |  |  |  |  |  |
| Yes | 273(39.7) |  | 42(5.0) | 278.909 | *p*<0.001*** |
| No | 414(60.3) |  | 799(95.0) |  |  |
| **Myalgia** |  |  |  |  |  |
| Yes | 165(24.0) |  | 57(6.8) | 90.499 | *p*<0.001*** |
| No | 522(76.0) |  | 784(93.2) |  |  |
| **Cough** |  |  |  |  |  |
| Yes | 84(12.2) |  | 27(3.2) | 45.632 | *p*<0.001*** |
| No | 603(87.8) |  | 814(96.8) |  |  |
| **Breathing difficulty** |  |  |  |  |  |
| Yes | 28(4.1) |  | 6(0.7) | 19.648 | *p*<0.001*** |
| No | 659(95.9) |  | 835(99.3) |  |  |
| **Dizziness** |  |  |  |  |  |
| Yes | 50(7.3) |  | 28(3.3) | 12.171 | *p*<0.001*** |
| No | 637(92.7) |  | 813(96.7) |  |  |
| **Coryza** |  |  |  |  |  |
| Yes | 74(10.8) |  | 45(5.4) | 15.471 | *p*<0.001*** |
| No | 613(89.2) |  | 796(94.6) |  |  |
| **Sore Throat** |  |  |  |  |  |
| Yes | 94(13.7) |  | 31(3.7) | 50.305 | *p*<0.001*** |
| No | 593(86.3) |  | 810(96.3) |  |  |
| **Persistent fever with cough or breathing difficulty** | | | | | |
| Yes | 3(0.4) |  | 1(0.1) | NA | NA |
| No | 684(99.6) |  | 840(99.9) |  |  |
| **Nausea, vomiting or diarrhea** |  |  |  |  |  |
| Yes | 49(7.1) |  | 7(0.8) | 42.509 | *p*<0.001*** |
| No | 638(92.9) |  | 834(99.2) |  |  |
| **Consultation with a doctor in an outpatient clinic in the last 14 days** | | | | | |
| Yes | 71(10.3) |  | 12(1.4) | 58.409 | *p*<0.001*** |
| No | 616(89.7) |  | 829(98.6) |  |  |
| **Hospitalization in the last 14 days** |  |  |  |  |  |
| Yes | 5(0.7) |  | 0(0) | 6.141 | *p*<0.05* |
| No | 682(99.3) |  | 841(100) |  |  |
| **Recent testing for COVID-19 in the past 14 days** | | | | | |
| Yes | 19(2.8) |  | 4(0.5) | 13.375 | *p*<0.001*** |
| No | 668(97.2) |  | 837(99.5) |  |  |
| **Recent quarantine in the past 14 days** | | | | | |
| Yes | 50(7.3) |  | 40(4.8) | 4.338 | *p*<0.05* |
| No | 637(92.7) |  | 801(95.2) |  |  |
| **Current self-rating of health status** |  |  |  |  |  |
| Very poor or poor | 4(0.6) |  | 13(1.5) | 103.132 | *p*<0.001*** |
| Average | 80(11.6) |  | 279(33.2) |  |  |
| Good or very good | 603(87.8) |  | 549(65.3) |  |  |
| **Chronic Illness** |  |  |  |  |  |
| Yes | 203(29.5) |  | 43(5.1) | 167.150 | *p*<0.001*** |
| No | 484(70.5) |  | 798(94.9) |  |  |
| **Direct contact with patients with a confirmed diagnosis of COVID-19** | | | | | |
| Yes | 111(16.2) |  | 2(0.2) | 139.925 | *p*<0.001*** |
| No | 576(83.8) |  | 839(99.8) |  |  |
| **Indirect contact with patients with a confirmed diagnosis of COVID-19** | | | | | |
| Yes | 110(16.0) |  | 6(0.7) | 126.143 | *p*<0.001*** |
| No | 577(84.0) |  | 835(99.3) |  |  |
| **Direct contact with materials contaminated by COVID-19** | | | | | |
| Yes | 55(8.0) |  | 4(0.5) | 57.758 | *p*<0.001*** |
| No | 632(92.0) |  | 837(99.5) |  |  |

* *p*<0.05, ***p*<0.01, ****p*<0.001, NA: due to too small number in one category and cannot perform Chi-square analysis

| **Table S4. Comparison of knowledge and concerns related to COVID-19 between Spanish and Chinese respondents (N= 1528)** | | | | | |
| --- | --- | --- | --- | --- | --- |
| **Knowledge and concerns related to COVID-19** | Spain  (N=687) |  | China  (N=841) | Chi-square (*χ^2^*) | *p*-value |
|  | N(%) |  | N(%) |  |  |
| **Route of transmission** |  |  |  |  |  |
| **Droplets** |  |  |  |  |  |
| Agree | 620(90.2) |  | 745(88.6) | 8.438 | *p*=0.015* |
| Disagree | 19(2.8) |  | 11(1.3) |  |  |
| Do not know | 48(7.0) |  | 85(10.1) |  |  |
| **Contact via contaminated objects** |  |  |  |  |  |
| Agree | 654(95.2) |  | 620(73.7) | 127.123 | *p*<0.001*** |
| Disagree | 13(1.9) |  | 56(6.7) |  |  |
| Do not know | 20(2.9) |  | 165(19.6) |  |  |
| **Airborne** |  |  |  |  |  |
| Agree | 301(43.8) |  | 487(57.9) | 90.307 | *p*<0.001*** |
| Disagree | 286(41.6) |  | 164(19.5) |  |  |
| Do not know | 100(14.6) |  | 190(22.6) |  |  |
| **Level of confidence in own doctor’s ability to diagnose or recognize COVID-19** | | | | | |
| Very confident | 362(52.7) |  | 485(57.7) | 27.275 | *p*<0.001*** |
| Somewhat confident | 263(38.3) |  | 318(37.8) |  |  |
| Not confident | 57(8.3) |  | 23(2.7) |  |  |
| Do not know | 5(0.7) |  | 15(1.8) |  |  |
| **Likelihood of contracting COVID−19 during the pandemic** | | | | | |
| Very likely | 112(16.3) |  | 77(9.2) | 133.557 | *p*<0.001*** |
| Somewhat likely | 268(39.0) |  | 227(27.0) |  |  |
| Not very likely | 276(40.2) |  | 328(39.0) |  |  |
| Not likely at all | 11(1.6) |  | 109(13.0) |  |  |
| Do not know | 20(2.9) |  | 100(11.8) |  |  |
| **Likelihood of surviving if infected with COVID-19** | | | | | |
| Very likely | 500(72.8) |  | 196(23.3) | 378.575 | *p*<0.001*** |
| Somewhat likely | 140(20.4) |  | 435(51.7) |  |  |
| Not very likely | 7(1.0) |  | 60(7.1) |  |  |
| Not likely at all | 2(0.3) |  | 14(1.7) |  |  |
| Do not know | 38(5.5) |  | 136(16.2) |  |  |
| **Satisfaction with the amount of health information available about COVID-19** | | | | | |
| Very satisfied | 223(32.5) |  | 243(28.9) |  |  |
| Somewhat satisfied | 305(44.4) |  | 508(60.4) |  |  |
| Not very satisfied | 122(17.7) |  | 43(5.1) | 82.549 | *p*<0.001*** |
| Not satisfied at all | 27(3.9) |  | 21(2.5) |  |  |
| Do not know | 10(1.5) |  | 26(3.1) |  |  |
| **Degree of worry about family members being diagnosed with COVID-19** | | | | | |
| Very worried | 318(46.3) |  | 276(32.8) | 103.231 | *p*<0.001*** |
| Fairly worried | 306(44.6) |  | 319(37.9) |  |  |
| Not very worried | 57(8.3) |  | 177(21.1) |  |  |
| Not worried at all | 5(0.7) |  | 56(6.7) |  |  |
| No family members | 1(0.1) |  | 13(1.5) |  |  |
| **After the outbreak, did you feel discriminated against by other countries?** | | | | | |
| Yes | 143(20.8) |  | 293(34.8) | 36.470 | *p*<0.001*** |
| No | 544(79.2) |  | 548(65.2) |  |  |
| **How much time do you spend monitoring information on the epidemic daily (hours)** | | | | | |
| [0-1] | 485(70.6) |  | 550(65.4) | 4.760 | *p*=0.093 |
| (1-3] | 170(24.7) |  | 242(28.8) |  |  |
| >3 | 32(4.7) |  | 49(5.8) |  |  |

* *p*<0.05, ***p*<0.01, ****p*<0.001

| **Table S5. Comparison of precautionary measures related to COVID-19 between Spanish and Chinese respondents (N= 1528)** | | | | | |
| --- | --- | --- | --- | --- | --- |
| **Precautionary Measures** | Spain  (N=687) |  | China  (N=841) | Chi-square (*χ^2^*) | *p*-value |
|  | N(%) |  | N(%) |  |  |
| **Covering mouth when coughing and sneezing** | | | | | |
| Always | 443(64.5) |  | 515(61.2) | 36.610 | *p*<0.001*** |
| Most of the time | 190(27.7) |  | 178(21.2) |  |  |
| Sometimes | 29(4.2) |  | 60(7.1) |  |  |
| Occasionally | 13(1.9) |  | 48(5.7) |  |  |
| Never | 12(1.7) |  | 40(4.8) |  |  |
| **Avoidance of sharing utensils (e.g., chopsticks) during meals** | | | | | |
| Always | 302(44.0) |  | 379(45.1) | 12.147 | *p*=0.016* |
| Most of the time | 172(25.0) |  | 153(18.2) |  |  |
| Sometimes | 85(12.4) |  | 127(15.1) |  |  |
| Occasionally | 61(8.9) |  | 87(10.3) |  |  |
| Never | 67(9.7) |  | 95(11.3) |  |  |
| **Washing hands with soap and water** | | | | | |
| Always | 545(79.3) |  | 488(58.0) | 97.808 | *p*<0.001*** |
| Most of the time | 111(16.2) |  | 204(24.3) |  |  |
| Sometimes | 14(2.0) |  | 86(10.2) |  |  |
| Occasionally | 5(0.7) |  | 38(4.5) |  |  |
| Never | 12(1.8) |  | 25(3.0) |  |  |
| **Washing hands immediately after coughing, rubbing the nose or sneezing** | | | | | |
| Always | 205(29.8) |  | 389(46.3) | 48.941 | *p*<0.001*** |
| Most of the time | 214(31.1) |  | 191(22.7) |  |  |
| Sometimes | 151(22.0) |  | 126(15.0) |  |  |
| Occasionally | 83(12.1) |  | 84(10.0) |  |  |
| Never | 34(5.0) |  | 51(6.0) |  |  |
| **Wearing a face mask regardless of the presence or absence of symptoms** | | | | | |
| Always | 182(26.5) |  | 619(73.6) | 516.487 | *p*<0.001*** |
| Most of the time | 95(13.8) |  | 154(18.3) |  |  |
| Sometimes | 95(13.8) |  | 44(5.2) |  |  |
| Occasionally | 72(10.5) |  | 16(1.9) |  |  |
| Never | 243(35.4) |  | 8(1.0) |  |  |
| **Washing hands after touching contaminated objects** | | | | | |
| Always | 502(73.1) |  | 624(74.2) | 18.134 | *P<*0.01** |
| Most of the time | 108(15.7) |  | 169(20.1) |  |  |
| Sometimes | 43(6.3) |  | 28(3.3) |  |  |
| Occasionally | 18(2.6) |  | 11(1.3) |  |  |
| Never | 16(2.3) |  | 9(1.1) |  |  |
| **Feeling that too much unnecessary worry has been made about the COVID-19 pandemic** | | | | | |
| Always | 14(2.1) |  | 123(14.7) | 315.008 | *p*<0.001*** |
| Most of the time | 126(18.3) |  | 80(9.5) |  |  |
| Sometimes | 310(45.1) |  | 155(18.4) |  |  |
| Occasionally | 158(23.0) |  | 128(15.2) |  |  |
| Never | 79(11.5) |  | 355(42.2) |  |  |

* *p*<0.05, ***p*<0.01, ****p*<0.001

| **Table S6. Comparison of information needs about COVID-19 between Spanish and Chinese respondents (N= 1528)** | | | | | | |  |
| --- | --- | --- | --- | --- | --- | --- | --- |
| **Information needs** | Spain  (N=687) |  | China  (N=841) | Chi-square (*χ^2^*) | | *p*-value |  |
|  | N(%) |  | N(%) |  |  |  |  |
| **Symptoms related to COVID-19** | | | | | | |  |
| Yes | 256(37.3) |  | 720(85.6) | 383.054 | | *p*<0.001*** |  |
| No | 431(62.7) |  | 121(14.4) |  |  |  |  |
| **Prevention methods** |  |  |  |  | |  | |
| Yes | 386(56.2) |  | 760(90.4) | 235.630 | | *p*<0.001*** |  |
| No | 301(43.8) |  | 81(9.6) |  |  |  |  |
| **Management and treatment methods** | | | | | | |  |
| Yes | 461(67.1) |  | 648(77.1) | 18.801 | | *p*<0.001*** |  |
| No | 226(32.9) |  | 193(22.9) |  |  |  |  |
| **Regular information updates** | | | | | | |  |
| Yes | 364(53.0) |  | 800(95.1) | 370.023 | | *p*<0.001*** |  |
| No | 323(47.0) |  | 41(4.9) |  |  |  |  |
| **More personalized information, such as COVID-19 and preexisting medical conditions** | | | | | | |  |
| Yes | 406(59.1) |  | 748(88.9) | 182.188 | | *p*<0.001*** |  |
| No | 281(40.9) |  | 93(11.1) |  |  |  |  |
| **Effectiveness of drugs and vaccines** | | | | | | |  |
| Yes | 528(76.9) |  | 781(92.9) | 78.933 | | *p*<0.001*** |  |
| No | 159(23.1) |  | 60(7.1) |  |  |  |  |
| **Number of infected by geographical location** | | | | | | |  |
| Yes | 288(41.9) |  | 778(92.5) | 458.737 | | *p*<0.001*** |  |
| No | 399(58.1) |  | 63(7.5) |  |  |  |  |
| **Travel advice** |  |  |  |  | |  |  |
| Yes | 306(44.5) |  | 742(88.2) | 334.946 | | *p*<0.001*** |  |
| No | 381(55.5) |  | 99(11.8) |  |  |  |  |
| **Transmission methods** |  |  |  |  | |  |  |
| Yes | 357(52.0) |  | 787(93.6) | 348.015 | | *p*<0.001*** |  |
| No | 330(48.0) |  | 54(6.4) |  |  |  |  |
| **Other countries’ strategies and responses** | | | | | | |  |
| Yes | 402(58.5) |  | 299(35.6) | | 80.295 | p<0.001*** |  |
| No | 285(41.5) |  | 542(64.4) | |  |  |  |

* *p*<0.05, ***p*<0.01, ****p*<0.001
