# Supplementary material for: The Impact of the COVID-19 Pandemic on Physical and Mental Health in China and Spain: Cross-sectional Study
Source: JMIR Form Res. 2021 May 21;5(5):e27818. doi: 10.2196/27818 (PMC8143872; doi:10.2196/27818)
Supplement: Multimedia Appendix 2 [file formative_v5i5e27818_app2.docx]

Multimedia Appendix 2

**Table S1.** Univariate linear regression analysis of the association between demographic characteristics and mental health parameters in Spanish and Chinese respondents adjusted for age, gender, and education (N=1528).

| **Demographic Characteristics** | **Spain** | | | | | | | |  | China | | | | | | | |
| --- | --- | --- | --- | --- | --- | --- | --- | --- | --- | --- | --- | --- | --- | --- | --- | --- | --- |
|  | **IES-R** | | **DASS-21**  **Stress** | | **DASS-21**  **Anxiety** | | **DASS-21**  **Depression** | |  | **IES-R** | | **DASS-21**  **Stress** | | **DASS-21**  **Anxiety** | | **DASS-21**  **Depression** | |
|  | ***B***(95%CI) | ***t*** | ***B***(95%CI) | ***t*** | *B*(95%CI) | *t* | *B*(95%CI) | *t* |  | *B*(95%CI) | *t* | *B*(95%CI) | *t* | *B*(95%CI) | *t* | *B*(95%CI) | *t* |
| **Gender** |  |  |  |  |  |  |  |  |  |  |  |  |  |  |  |  |  |
| Male | -0.40  (-0.64, -0.15) | -3.23** | -0.30  (-0.50, -0.09) | -2.87** | -0.15 | -1.17 | -0.27  (-0.48, -0.05) | -2.40* |  | -0.26  (-0.46, -0.06) | -2.55* | 0.09 | 1.57 | 0.20  (0.02, 0.38) | 2.16* | 0.24  (0.09, 0.39) | 3.17** |
| Female | Reference=0 | | Reference=0 | | Reference=0 | | Reference=0 | |  | Reference=0 | | Reference=0 | | Reference=0 | | Reference=0 | |
| **Age range** |  |  |  |  |  |  |  |  |  |  |  |  |  |  |  |  |  |
| [18-21]† | 0.85  (0.33, 1.38) | 3.19** | 0.85  (0.41, 1.28) | 3.81*** | 0.67  (0.12, 1.22) | 2.38* | 0.74  (0.27, 1.22) | 3.07** |  | 0.78  (0.12, 1.45) | 2.32* | -0.05 | -0.24 | 0.26 | 0.83 | -0.05 | -0.20 |
| [22-30] | 0.39  (0.07, 0.71) | 2.37* | 0.62  (0.36, 0.89) | 4.58*** | 0.21 | 1.19 | 0.41  (0.12, 0.70) | 2.78** |  | 0.59 | 1.75 | 0.02 | 0.08 | 0.36 | 1.18 | 0.09 | 0.36 |
| [31-40] | 0.42  (0.15, 0.69) | 3.03** | 0.62  (0.39, 0.84) | 5.42*** | 0.49  (0.20, 0.77) | 3.37** | 0.36  (0.11, 0.60) | 2.87** |  | 0.63 | 1.62 | -0.03 | -0.15 | 0.29 | 0.81 | 0.03 | 0.12 |
| [41-49] | 0.29  (0.04, 0.55) | 2.27* | 0.36  (0.15, 0.57) | 3.34** | 0.29  (0.03, 0.56) | 2.15* | 0.18 | 1.50 |  | 0.26 | 0.70 | -0.15 | -0.70 | -0.02 | -0.05 | -0.18 | -0.64 |
| ≥50 | Reference=0 | | Reference=0 | | Reference=0 | | Reference=0 | |  | Reference=0 | | Reference=0 | | Reference=0 | | Reference=0 | |
| **Marital status** |  |  |  |  |  |  |  |  |  |  |  |  |  |  |  |  |  |
| Never married | -0.07 | -0.17 | 0.05 | 0.16 | 0.33 | 0.77 | -0.16 | -0.43 |  | 1.06 | 1.42 | 0.41 | 0.94 | 0.60 | 0.87 | 0.48 | 0.85 |
| Married | -0.06 | -0.14 | 0.28 | 0.77 | 0.87 | 1.95 | 0.15 | 0.40 |  | 1.28 | 1.72 | 0.45 | 1.05 | 0.80 | 1.16 | 0.56 | 1.01 |
| Divorcee | 0.19 | 0.47 | 0.38 | 1.11 | 0.51 | 1.21 | 0.22 | 0.61 |  | 1.27 | 1.35 | 0.60 | 1.10 | 1.00 | 1.17 | 0.60 | 0.85 |
| Widowhood | Reference=0 | | Reference=0 | | Reference=0 | | Reference=0 | |  | Reference=0 | | Reference=0 | | Reference=0 | | Reference=0 | |
| **Parental status** |  |  |  |  |  |  |  |  |  |  |  |  |  |  |  |  |  |
| Has a child 16 years or below | 0.37  （0.11,0.62） | 2.85** | 0.49  (0.28, 0.70) | 4.55*** | 0.26 | 1.91 | 0.46  (0.23, 0.69) | 3.94*** |  | 0.12 | 0.93 | 0.02 | 0.28 | 0.09 | 0.76 | 0.08 | 0.78 |
| Has a child older than 16 years | 0.33  (0.06, 0.60) | 2.36* | 0.50  (0.27, 0.72) | 4.24*** | 0.33  (0.04, 0.62) | 2.23* | 0.25 | 1.96 |  | 0.12 | 1.21 | 0.01 | 0.17 | -0.03 | -0.29 | -0.02 | -0.20 |
| No children | Reference=0 | | Reference=0 | | Reference=0 | | Reference=0 | |  | Reference=0 | | Reference=0 | | Reference=0 | | Reference=0 | |
| **Family size** |  |  |  |  |  |  |  |  |  |  |  |  |  |  |  |  |  |
| 6 people or more | 0.69 | 1.34 | 0.36 | 0.83 | 0.67 | 1.24 | 0.08 | 0.18 |  | 1.43  (0.14, 2.71) | 2.18* | 0.50 | 1.32 | 0.85 | 1.43 | 0.13 | 0.26 |
| 3-5 people | -0.04 | -0.27 | 0.003 | 0.02 | -0.11 | -0.70 | -0.13 | -1.02 |  | 1.32  (0.05, 2.59) | 2.05* | 0.44 | 1.17 | 0.75 | 1.28 | 0.05 | 0.10 |
| 2 people | -0.22 | -1.40 | -0.12 | -0.90 | -0.28 | -1.67 | -0.25 | -1.74 |  | 1.19 | 1.77 | 0.44 | 1.13 | 0.61 | 0.99 | -0.16 | -0.31 |
| 1 person | Reference=0 | | Reference=0 | | Reference=0 | | Reference=0 | |  | Reference=0 | | Reference=0 | | Reference=0 | | Reference=0 | |
| **Level of education** |  |  |  |  |  |  |  |  |  |  |  |  |  |  |  |  |  |
| High School | 0.62 | 1.64 | 0.50 | 1.57 | 0.54 | 1.35 | 0.58 | 1.70 |  | 0.05 | 0.18 | 0.30 | 1.87 | 0.38 | 1.53 | 0.17 | 0.84 |
| University – Bachelor | 0.47 | 1.26 | 0.26 | 0.81 | 0.15 | 0.38 | 0.21 | 0.63 |  | 0.44  (0.08, 0.80) | 2.41* | 0.16 | 1.46 | 0.16 | 0.95 | 0.10 | 0.69 |
| University – Master or Doctorate | 0.37 | 0.99 | 0.35 | 1.12 | 0.12 | 0.31 | 0.26 | 0.78 |  | 0.35 | 1.63 | 0.23 | 1.83 | 0.37 | 1.89 | 0.24 | 1.53 |
| Secondary School and below | Reference=0 | | Reference=0 | | Reference=0 | | Reference=0 | |  | Reference=0 | | Reference=0 | | Reference=0 | | Reference=0 | |

**P<*.05, ***P<*.01, ****P<*.001.

B: unstandardized coefficient.

**Table S2.** Univariate linear regression analysis of the association between symptoms related to COVID-19, health status, and contact history and mental health parameters in Spanish and Chinese respondents adjusted for age, gender, and education (N=1528).

| **Variable** | Spain | | | | | | | |  | China | | | | | | | | | | | | | | |
| --- | --- | --- | --- | --- | --- | --- | --- | --- | --- | --- | --- | --- | --- | --- | --- | --- | --- | --- | --- | --- | --- | --- | --- | --- |
|  | IES-R | | DASS-21  Stress | | DASS-21  Anxiety | | DASS-21  Depression | |  | IES-R | | | | DASS-21  Stress | | | | DASS-21  Anxiety | | | | | DASS-21  Depression | |
|  | *B*(95%CI) | *t* | *B*(95%CI) | *t* | *B*(95%CI) | *t* | *B*(95%CI) | *t* |  | *B*(95%CI) | | *t* | | *B*(95%CI) | | *t* | | *B*(95%CI) | | *t* | | | *B*(95%CI) | *t* |
| **Persistent fever** |  |  |  |  |  |  |  |  |  |  | |  | |  | |  | |  | |  | | |  |  |
| Yes | -0.03 | -0.09 | -0.20 | -0.68 | 0.08 | 0.21 | -0.45 | -1.42 |  | 1.45 | | 1.13 | | 3.53  (2.08, 4.97) | | 4.78*** | | 3.13  (0.84, 5.43) | | 2.69** | | | 3.34  (1.47, 5.22) | 3.50*** |
| No | Reference=0 | | Reference=0 | | Reference=0 | | Reference=0 | |  | Reference=0 | | | | Reference=0 | | | | Reference=0 | | | | | Reference=0 | |
| **Chills** |  |  |  |  |  |  |  |  |  |  | |  | |  | |  | |  | |  | | |  |  |
| Yes | 0.04 | 0.20 | 0.01 | 0.09 | 0.04 | 0.18 | 0.10 | 0.63 |  | 0.53 | | 1.36 | | 0.76  (0.32, 1.20) | | 3.37** | | 0.63 | | 1.79 | | | 0.67  (0.10, 1.24) | 2.31* |
| No | Reference=0 | | Reference=0 | | Reference=0 | | Reference=0 | |  | Reference=0 | | | | Reference=0 | | | | Reference=0 | | | | | Reference=0 | |
| **Headache** |  |  |  |  |  |  |  |  |  |  | |  | |  | |  | |  | |  | | |  |  |
| Yes | 0.32  (0.12, 0.52) | 3.07** | 0.28  (0.11, 0.45) | 3.23** | 0.40  (0.19, 0.62) | 3.69*** | 0.32  (0.14, 0.50) | 3.42** |  | 0.48  (0.08, 0.88) | | 2.38* | | 0.48  (0.25, 0.71) | | 4.10*** | | 0.81  (0.45, 1.17) | | 4.39*** | | | 0.52  (0.23, 0.82) | 3.46** |
| No | Reference=0 | | Reference=0 | | Reference=0 | | Reference=0 | |  | Reference=0 | | | | Reference=0 | | | | Reference=0 | | | | | Reference=0 | |
| **Myalgia** |  |  |  |  |  |  |  |  |  |  | |  | |  | |  | |  | |  | | |  |  |
| Yes | 0.47  (0.24, 0.70) | 4.04*** | 0.38  (0.19, 0.57) | 3.90*** | 0.39  (0.15, 0.63) | 3.16** | 0.25  (0.04, 0.45) | 2.33* |  | 0.48  (0.13, 0.82) | | 2.72** | | 0.42  (0.22, 0.62) | | 4.17*** | | 0.59  (0.28, 0.90) | | 3.70*** | | | 0.59  (0.33, 0.85) | 4.53*** |
| No | Reference=0 | | Reference=0 | | Reference=0 | | Reference=0 | |  | Reference=0 | | | | Reference=0 | | | | Reference=0 | | | | | Reference=0 | |
| **Cough** |  |  |  |  |  |  |  |  |  |  | |  | |  | |  | |  | |  | | |  |  |
| Yes | 0.30 | 1.95 | 0.03 | 0.21 | 0.20 | 1.25 | 0.07 | 0.48 |  | 0.57  (0.08, 1.06) | | 2.27* | | 0.63  (0.35, 0.92) | | 4.38*** | | 0.70  (0.26, 1.15) | | 3.09** | | | 0.69  (0.33, 1.06) | 3.72*** |
| No | Reference=0 | | Reference=0 | | Reference=0 | | Reference=0 | |  | Reference=0 | | | | Reference=0 | | | | Reference=0 | | | | | Reference=0 | |
| **Breathing difficulty** |  |  |  |  |  |  |  |  |  |  | |  | |  | |  | |  | |  | | |  |  |
| Yes | 0.41 | 1.61 | 0.11 | 0.50 | 0.60  (0.08, 1.12) | 2.27* | 0.32 | 1.42 |  | 0.78 | | 1.48 | | 1.03  (0.43, 1.63) | | 3.39** | | 0.98  (0.04, 1.92) | | 2.05* | | | 1.39  (0.62, 2.16) | 3.55*** |
| No | Reference=0 | | Reference=0 | | Reference=0 | | Reference=0 | |  | Reference=0 | | | | Reference=0 | | | | Reference=0 | | | | | Reference=0 | |
| **Dizziness** |  |  |  |  |  |  |  |  |  |  | |  | |  | |  | |  | |  | | |  |  |
| Yes | 0.42  (0.05, 0.80) | 2.22* | 0.32  (0.01, 0.63) | 2.01* | 0.60  (0.20, 0.99) | 2.97** | 0.42  (0.08, 0.76) | 2.43* |  | 0.87  (0.39, 1.35) | | 3.55*** | | 0.74  (0.46, 1.02) | | 5.21*** | | 0.82  (0.38, 1.26) | | 3.67*** | | | 0.50  (0.14, 0.86) | 2.72** |
| No | Reference=0 | | Reference=0 | | Reference=0 | | Reference=0 | |  | Reference=0 | | | | Reference=0 | | | | Reference=0 | | | | | Reference=0 | |
| **Coryza** |  |  |  |  |  |  |  |  |  |  | |  | |  | |  | |  | |  | | |  |  |
| Yes | 0.20 | 1.22 | 0.19 | 1.45 | 0.21 | 1.22 | -0.06 | -0.38 |  | 0.64  (0.25, 1.03) | | 3.25** | | 0.34  (0.11, 0.56) | | 2.92** | | 0.52  (0.16, 0.87) | | 2.87** | | | 0.55  (0.26, 0.84) | 3.76*** |
| No | Reference=0 | | Reference=0 | | Reference=0 | | Reference=0 | |  | Reference=0 | | | | Reference=0 | | | | Reference=0 | | | | | Reference=0 | |
| **Sore Throat** |  |  |  |  |  |  |  |  |  |  | |  | |  | |  | |  | |  | | |  |  |
| Yes | 0.36  (0.07, 0.64) | 2.47* | 0.30  (0.06, 0.53) | 2.47* | 0.61  (0.31, 0.90) | 4.01*** | 0.28  (0.03, 0.54) | 2.17* |  | 0.31 | | 1.31 | | 0.59  (0.33, 0.86) | | 4.38*** | | 0.76  (0.34, 1.18) | | 3.56*** | | | 0.71  (0.37, 1.05) | 4.07*** |
| No | Reference=0 | | Reference=0 | | Reference=0 | | Reference=0 | |  | Reference=0 | | | | Reference=0 | | | | Reference=0 | | | | | Reference=0 | |
| **Persistent fever with cough or breathing difficulty** | | | | | | |  |  |  |  | |  | |  | |  | |  | |  | | |  |  |
| Yes | -0.61 | -0.81 | -0.82 | -1.30 | -0.83 | -1.04 | -0.81 | -1.18 |  | 1.45 | | 1.13 | | 3.53  (2.08,4.97) | | 4.78*** | | 3.13  (0.84, 5.43) | | 2.69** | | | 3.34  (1.47, 5.22) | 3.50*** |
| No | Reference=0 | | Reference=0 | | Reference=0 | | Reference=0 | |  | Reference=0 | | | | Reference=0 | | | | Reference=0 | | | | | Reference=0 | |
| **Nausea, vomiting or diarrhea** |  |  |  |  |  |  |  |  |  |  | |  | |  | |  | |  | |  | | |  |  |
| Yes | 0.64  (0.26, 1.02) | 3.32** | 0.46  (0.15, 0.78) | 2.89** | 0.89  (0.49, 1.28) | 4.41*** | 0.47  (0.13, 0.81) | 2.70** |  | 0.70 | | 1.44 | | 1.27  (0.72, 1.82) | | 4.52*** | | 1.37  (0.50, 2.24) | | 3.10** | | | 1.28  (0.57, 1.99) | 3.53*** |
| No | Reference=0 | | Reference=0 | | Reference=0 | | Reference=0 | |  | Reference=0 | | | | Reference=0 | | | | Reference=0 | | | | | Reference=0 | |
| **Doctor consultation in the past 2 weeks** | | | | | | | | |  |  | |  | |  | |  | |  | |  | | |  |  |
| Yes | 0.40  (0.07, 0.72) | 2.41* | 0.17 | 1.21 | 0.53  (0.20, 0.87) | 3.11** | 0.09 | 0.59 |  | 0.61 | | 1.64 | | 0.19 | | 0.87 | | 0.25 | | 0.74 | | | 0.12 | 0.43 |
| No | Reference=0 | | Reference=0 | | Reference=0 | | Reference=0 | |  | Reference=0 | | | | Reference=0 | | | | Reference=0 | | | | | Reference=0 | |
| **Recent COVID-19 testing in the past 2 weeks** | | | | | | | | |  |  | |  | |  | |  | |  | |  | | |  |  |
| Yes | 0.41 | 1.35 | -0.04 | -0.14 | 0.62 | 1.93 | -0.01 | -0.02 |  | -0.42 | | -0.65 | | -0.20 | | -0.53 | | -0.03 | | -0.06 | | | -0.05 | -0.10 |
| No | Reference=0 | | Reference=0 | | Reference=0 | | Reference=0 | |  | Reference=0 | | | | Reference=0 | | | | Reference=0 | | | | | Reference=0 | |
| **Recent quarantine in the past 2 weeks** | | | | | | | | |  |  | |  | |  | |  | |  | |  | | |  |  |
| Yes | 0.43  (0.05, 0.80) | 2.23* | 0.06 | 0.35 | 0.52  (0.13, 0.92) | 2.60* | 0.03 | 0.17 |  | 0.31 | | 1.49 | | 0.19 | | 1.60 | | 0.48  (0.11, 0.85) | | 2.52* | | | 0.32  (0.01, 0.62) | 2.03* |
| No | Reference=0 | | Reference=0 | | Reference=0 | | Reference=0 | |  | Reference=0 | | | | Reference=0 | | | | Reference=0 | | | | | Reference=0 | |
| **Health status (Self-ratting)** |  |  |  |  |  |  |  |  |  |  | |  | |  | |  | |  | |  | | |  |  |
| Very poor or poor | 1.15 | 1.79 | 1.75  (0.70, 2.79) | 3.26** | 1.89  (0.57, 3.21) | 2.81** | 1.81  (0.67, 2.94) | 3.13** |  | 0.53 | | 1.47 | | 0.83  (0.42, 1.23) | | 4.02*** | | 1.13  (0.50, 1.77) | | 3.52*** | | | 0.84  (0.33, 1.36) | 3.19** |
| Average | 0.69  (0.39, 0.99) | 4.44*** | 0.51  (0.26, 0.76) | 3.96*** | 0.88  (0.56, 1.19) | 5.41*** | 0.69  (0.41, 0.96) | 4.94*** |  | 0.40  (0.22, 0.59) | | 4.24*** | | 0.24  (0.14, 0.35) | | 4.48*** | | 0.45  (0.28, 0.62) | | 5.29*** | | | 0.41  (0.27, 0.54) | 5.83*** |
| Good or very good | Reference=0 | | Reference=0 | | Reference=0 | | Reference=0 | |  | Reference=0 | | | | Reference=0 | | | | Reference=0 | | | | | Reference=0 | |
| **Past medical illness** |  | |  | |  | |  | |  |  | | | |  | | | |  | | | | |  | |
| Yes | 0.24  (0.03, 0.46) | 2.22* | 0.17 | 1.82 | 0.35  (0.12, 0.58) | 3.03** | 0.17 | 1.67 |  | 0.41  (0.01, 0.80) | | 2.02* | | 0.25  (0.02, 0.48) | | 2.10* | | 0.28 | | 1.51 | | | 0.29 | 1.95 |
| No | Reference=0 | | Reference=0 | | Reference=0 | | Reference=0 | |  | Reference=0 | | | | Reference=0 | | | | Reference=0 | | | | | Reference=0 | |
| **Direct contact with a confirmed case of COVID-19** | | | | | | | | |  |  | |  | |  | |  | |  | |  | | |  |  |
| Yes | 0.13 | 0.94 | 0.07 | 0.62 | 0.15 | 1.04 | -0.04 | -0.29 |  | -1.52 | | -1.67 | | 0.21 | | 0.39 | | 0.51 | | 0.61 | | | 0.69 | 1.00 |
| No | Reference=0 | | Reference=0 | | Reference=0 | | Reference=0 | |  | Reference=0 | | | Reference=0 | | | | Reference=0 | | | | Reference=0 | | | |
| **Indirect contact with a confirmed case of COVID-19** | | | | | | | | |  |  | |  | |  | |  | |  | |  | | |  |  |
| Yes | -0.26 | -1.89 | -0.06 | -0.49 | -0.29  (-0.57, -0.01) | -2.05* | -0.19 | -1.55 |  | -0.32 | | -0.60 | | 0.13 | | 0.43 | | 0.22 | | 0.45 | | | 0.26 | 0.65 |
| No | Reference=0 | | Reference=0 | | Reference=0 | | Reference=0 | |  | | Reference=0 | | | | Reference=0 | | | | Reference=0 | | | Reference=0 | | |
| **Contact with materials contaminated by COVID-19** | | | | | | | | |  |  | |  | |  | |  | |  | |  | | |  |  |
| Yes | 0.06 | 0.34 | -0.08 | -0.55 | -0.11 | -0.57 | -0.18 | -1.11 |  | -1.10 | | -1.71 | | 0.13 | | 0.35 | | 0.36 | | 0.61 | | | 0.32 | 0.67 |
| No | Reference=0 | | Reference=0 | | Reference=0 | | Reference=0 | |  | Reference=0 | | | | Reference=0 | | | | Reference=0 | | | | | Reference=0 | |

**P<*.05, ***P<*.01, ****P<*.001.

B: unstandardized coefficient.

**Table S3.** Univariate linear regression analysis of the association of views and knowledge of COVID-19 and mental health parameters between Spanish and Chinese respondents adjusted for age, gender, and education (N=1528).

|  | Spain | | | | | | | |  | China | | | | | | | |
| --- | --- | --- | --- | --- | --- | --- | --- | --- | --- | --- | --- | --- | --- | --- | --- | --- | --- |
| **Views and Knowledge on COVID-19** | IES-R | | DASS-21  Stress | | DASS-21  Anxiety | | DASS-21  Depression | |  | IES-R | | DASS-21  Stress | | DASS-21  Anxiety | | DASS-21  Depression | |
|  | *B*  (95%CI) | *t* | *B*  (95%CI) | *t* | *B*  (95%CI) | *t* | *B*  (95%CI) | *t* |  | *B*  (95%CI) | *t* | *B*  (95%CI) | *t* | *B*  (95%CI) | *t* | *B*  (95%CI) | *t* |
| **Transmission by respiratory droplets** | | | | | | | | |  |  |  |  |  |  |  |  |  |
| Agree | 0.33 | 1.67 | 0.21 | 1.27 | 1.45 | 0.70 | 0.18 | 1.03 |  | -0.01 | -0.08 | -0.14 | -1.57 | -0.15 | -1.08 | -0.17 | -1.49 |
| Disagree | 0.30 | 0.86 | 0.42 | 1.42 | 0.37 | 0.98 | 0.17 | 0.53 |  | 0.05 | 0.11 | 0.43 | 1.80 | 0.66 | 1.77 | 0.50 | 1.62 |
| Do not know | Reference=0 | | Reference=0 | | Reference=0 | | Reference=0 | |  | Reference=0 | | Reference=0 | | Reference=0 | | Reference=0 | |
| **Transmission by touching contaminated objects** | | | | | | | | |  |  |  |  |  |  |  |  |  |
| Agree | 0.30 | 1.00 | -0.04 | -0.17 | 0.32 | 1.03 | 0.20 | 0.76 |  | -0.08 | -0.71 | -0.09 | -1.35 | -0.06 | -0.56 | -0.11 | -1.26 |
| Disagree | -0.41 | -0.87 | -0.57 | -1.47 | -0.61 | -1.25 | -0.38 | -0.90 |  | 0.05 | 0.25 | -0.02 | -0.19 | -0.18 | -0.99 | -0.17 | -1.17 |
| Do not know | Reference=0 | | Reference=0 | | Reference=0 | | Reference=0 | |  | Reference=0 | | Reference=0 | | Reference=0 | | Reference=0 | |
| **Airborne transmission** |  |  |  |  |  |  |  |  |  |  |  |  |  |  |  |  |  |
| Agree | -0.26 | -1.74 | -0.20 | -1.56 | -0.38  (-0.70, -0.07) | -2.40* | -0.14 | -1.00 |  | 0.03 | 0.30 | 0.02 | 0.25 | 0.01 | 0.08 | -0.07 | -0.78 |
| Disagree | -0.24 | -1.59 | -0.13 | -1.04 | -0.36  (-0.68, -0.05) | -2.27* | -0.16 | -1.13 |  | -0.05 | -0.36 | -0.05 | -0.64 | -0.05 | -0.42 | -0.08 | -0.76 |
| Do not know | Reference=0 | | Reference=0 | | Reference=0 | | Reference=0 | |  | Reference=0 | | Reference=0 | | Reference=0 | | Reference=0 | |
| **Level of confidence in the competency of doctors to diagnose COVID-19** | | | | | | | | |  |  |  |  |  |  |  |  |  |
| Very confident | -0.39 | -0.66 | -0.50 | -1.01 | -0.99 | -1.58 | -0.67 | -1.25 |  | -0.07 | -0.22 | -0.28 | -1.40 | -0.35 | -1.12 | -0.79  (-1.29, -0.29) | -3.11** |
| Somewhat confident | -0.27 | -0.45 | -0.38 | -0.77 | -0.97 | -1.55 | -0.42 | -0.78 |  | 0.23 | 0.68 | -0.19 | -0.93 | -0.22 | -0.71 | -0.64  (-1.14, -0.14) | -2.49* |
| Little confident | -0.01 | -0.02 | -0.20 | -0.39 | -0.79 | -1.22 | -0.54 | -0.97 |  | 0.30 | 0.70 | 0.05 | 0.21 | 0.11 | 0.29 | -0.41 | -1.26 |
| No confidence | -0.15 | -0.19 | -0.65 | -0.99 | -1.03 | -1.23 | 0.18 | 0.25 |  | -0.19 | -0.19 | -0.24 | -0.42 | 0.53 | 0.60 | -0.22 | -0.31 |
| Do not know | Reference=0 | | Reference=0 | | Reference=0 | | Reference=0 | |  | Reference=0 | | Reference=0 | | Reference=0 | | Reference=0 | |
| **Perceived risk of contracting COVID−19 during the pandemic** | | | | | | | | |  |  |  |  |  |  |  |  |  |
| Very high risk | 0.48 | 1.51 | 0.20 | 0.74 | 0.46 | 1.37 | 0.26 | 0.91 |  | -0.20 | -1.01 | -0.08 | -0.71 | 0.06 | 0.31 | -0.08 | -0.52 |
| High risk | 0.29 | 0.95 | 0.22 | 0.85 | 0.46 | 1.43 | 0.24 | 0.89 |  | -0.02 | -0.13 | -0.14 | -1.54 | -0.02 | -0.14 | -0.12 | -1.02 |
| Low risk | 0.18 | 0.58 | 0.12 | 0.47 | 0.24 | 0.75 | 0.22 | 0.80 |  | 0.07 | 0.47 | -0.15 | -1.71 | -0.07 | -0.50 | -0.12 | -1.08 |
| No risk | 0.51 | 1.03 | 0.23 | 0.56 | 0.61 | 1.19 | 0.66 | 1.49 |  | -0.12 | -0.65 | -0.21  (-0.41,-0.002) | -1.98* | -0.13 | -0.80 | -0.28  (-0.55, -0.02) | -2.11* |
| Do not know | Reference=0 | | Reference=0 | | Reference=0 | | Reference=0 | |  | Reference=0 | | Reference=0 | | Reference=0 | | Reference=0 | |
| **Perceived chance of survival after COVID-19 infection** | | | | | | | | |  |  |  |  |  |  |  |  |  |
| Very high chance of survival | 0.01 | 0.05 | -0.14 | -0.76 | -0.21 | -0.90 | -0.20 | -0.99 |  | -0.30  (-0.58, -0.01) | -2.06* | -0.16 | -1.85 | -0.18 | -1.37 | -0.25  (-0.46, -0.04) | -2.32* |
| High chance of survival | 0.41 | 1.73 | 0.29 | 1.47 | 0.30 | 1.18 | 0.18 | 0.85 |  | -0.02 | -0.15 | -0.07 | -0.93 | -0.06 | -0.52 | -0.16 | -1.72 |
| Low chance of survival | 0.85 | 1.59 | 0.42 | 0.96 | 0.52 | 0.92 | 0.54 | 1.11 |  | 0.03 | 0.17 | 0.05 | 0.41 | 0.40  (0.04, 0.75) | 2.19* | 0.19 | 1.27 |
| Very low chance of survival | -1.08 | -1.13 | -1.02 | -1.29 | -0.85 | -0.84 | -0.85 | -0.98 |  | -0.36 | -0.99 | 0.05 | 0.23 | 0.38 | 1.15 | 0.18 | 0.68 |
| Do not know | Reference=0 | | Reference=0 | | Reference=0 | | Reference=0 | |  | Reference=0 | | Reference=0 | | Reference=0 | | Reference=0 | |
| **Satisfaction with health information about COVID-19** | | | | | | | | |  |  |  |  |  |  |  |  |  |
| Very high level of satisfaction | 0.14 | 0.34 | 0.16 | 0.46 | 0.04 | 0.10 | 0.16 | 0.43 |  | -0.11 | -0.41 | -0.61  (-0.91, -0.31) | -4.03*** | -0.71  (-1.18, -0.24) | -2.95** | -0.74  (-1.13, -0.36) | -3.79*** |
| High level of satisfaction | 0.31 | 0.74 | 0.26 | 0.75 | 0.19 | 0.44 | 0.31 | 0.81 |  | 0.29 | 1.13 | -0.48  (-0.77, -0.19) | -3.23** | -0.60  (-1.06, -0.14) | -2.57* | -0.61  (-0.99, -0.23) | -3.16** |
| Low level of satisfaction | 0.65 | 1.53 | 0.48 | 1.35 | 0.31 | 0.69 | 0.38 | 0.97 |  | 0.08 | 0.25 | -0.36 | -1.95 | -0.49 | -1.68 | -0.38 | -1.59 |
| Not satisfied at all | 0.88 | 1.84 | 0.66 | 1.63 | 0.64 | 1.25 | 0.53 | 1.22 |  | 0.80  (0.07, 1.54) | 2.16* | 0.06 | 0.26 | 0.17 | 0.50 | -0.19 | -0.67 |
| Do not know | Reference=0 | | Reference=0 | | Reference=0 | | Reference=0 | |  | Reference=0 | | Reference=0 | | Reference=0 | | Reference=0 | |
| **Degree of worry about family members being diagnosed with COVID-19** | | | | | | | | |  |  |  |  |  |  |  |  |  |
| Very worried | 1.64 | 1.33 | 1.53 | 1.45 | 1.48 | 1.11 | 1.24 | 1.06 |  | -0.05 | -0.14 | -0.52  (-0.94, -0.11) | -2.50* | -0.31 | -0.92 | -0.73  (-1.26, -0.19) | -2.68** |
| Fairly worried | 0.91 | 0.73 | 1.10 | 1.04 | 0.83 | 0.62 | 0.94 | 0.80 |  | 0.01 | 0.03 | -0.65  (-1.06, -0.23) | -3.07** | -0.34 | -1.03 | -0.77  (-1.30, -0.23) | -2.82** |
| Not very worried | 0.25 | 0.20 | 0.56 | 0.53 | 0.43 | 0.32 | 0.43 | 0.36 |  | -0.39 | -1.06 | -0.76  (-1.18, -0.34) | -3.57*** | -0.60 | -1.80 | -0.91  (-1.45, -0.37) | -3.31** |
| Not worried at all | 0.54 | 0.40 | 0.98 | 0.85 | 0.91 | 0.62 | 1.39 | 1.09 |  | -0.56 | -1.43 | -0.73  (-1.17, -0.28) | -3.19** | -0.62 | -1.73 | -0.91  (-1.49, -0.33) | -3.09** |
| No family members | Reference=0 | | Reference=0 | | Reference=0 | | Reference=0 | |  | Reference=0 | | Reference=0 | | Reference=0 | | Reference=0 | |
| **How much time do you spend monitoring information on the pandemic daily (hours)** | | | | | | | | |  |  |  |  |  |  |  |  |  |
| ≦1 | -0.54  (-1.01, -0.08) | -2.30* | -0.48  (-0.87, -0.09) | -2.44* | -0.79  (-1.28, -0.31) | -3.19** | -0.54  (-0.97, -0.12) | -2.54* |  | 0.05 | 0.39 | 0.06 | 0.77 | -0.03 | -0.29 | -0.04 | -0.37 |
| （1h-3h] | -0.17 | -0.69 | -0.30 | -1.42 | -0.48 | -1.83 | -0.33 | -1.47 |  | -0.05 | -0.50 | -0.07 | -1.14 | -0.10 | -1.05 | -0.06 | -0.73 |
| ﹥3 | Reference=0 | | Reference=0 | | Reference=0 | | Reference=0 | |  | Reference=0 | | Reference=0 | | Reference=0 | | Reference=0 | |
| **After the outbreak, did you feel discriminated against by other countries?** | | | | | | | | |  |  |  |  |  |  |  |  |  |
| Yes | 0.45  (0.21, 0.69) | 3.68*** | 0.36  (0.16, 0.56) | 3.57*** | 0.38  (0.13, 0.64) | 2.99** | 0.28  (0.07, 0.50) | 2.57* |  | 0.17 | 1.86 | 0.10 | 1.83 | 0.08 | 0.93 | 0.08 | 1.19 |
| No | Reference=0 | | Reference=0 | | Reference=0 | | Reference=0 | |  | Reference=0 | | Reference=0 | | Reference=0 | | Reference=0 | |

**P<*.05, ***P<*.01, ****P<*.001.

B: unstandardized coefficient.

**Table S4.** Univariate linear regression analysis of the association between precautionary measures and mental health parameters in Spanish and Chinese participants adjusted for age, gender, and education (N=1528).

| **Precautionary Measures** | Spain | | | | | | | |  | China | | | | | | | |
| --- | --- | --- | --- | --- | --- | --- | --- | --- | --- | --- | --- | --- | --- | --- | --- | --- | --- |
|  | IES-R | | DASS-21  Stress | | DASS-21  Anxiety | | DASS-21  Depression | |  | IES-R | | DASS-21  Stress | | DASS-21  Anxiety | | DASS-21  Depression | |
|  | *B*  (95%CI) | *t* | *B*  (95%CI) | *t* | *B*  (95%CI) | *t* | *B*  (95%CI) | *t* |  | *B*  (95%CI) | *t* | *B*  (95%CI) | *t* | *B*  (95%CI) | *t* | *B*  (95%CI) | *t* |
| **Covering mouth when the participants coughed and sneezed** | | | | | | | | |  |  |  |  |  |  |  |  |  |
| Always | -0.48 | -1.25 | -0.72  (-1.35, -0.10) | -2.27* | -0.93  (-1.72, -0.13) | -2.30* | -0.53 | -1.53 |  | 0.10 | 0.50 | -0.32  (-0.56,-0.08) | -2.60** | -0.50  (--0.88,-0.13) | -2.62** | -0.56  (-0.87, -0.25) | -3.57*** |
| Most of the time | -0.44 | -1.12 | -0.67  (-1.30, -0.03) | -2.06* | -0.88  (-1.68, -0.07) | -2.14* | -0.43 | -1.23 |  | 0.37 | 1.66 | -0.33  (-0.59,-0.08) | -2.56* | -0.45  (-0.85, -0.05) | -2.21* | -0.43  (-0.76, -0.10) | -2.59* |
| Sometimes | -0.14 | -0.32 | -0.62 | -1.68 | -0.76 | -1.61 | -0.23 | -0.56 |  | 0.51 | 1.96 | -0.23 | -1.52 | -0.48  (-0.95, -0.01) | -2.02* | -0.40  (-0.78, -0.02) | -2.07* |
| Occasionally | -0.48 | -0.92 | -0.46 | -1.06 | -1.17  (-2.25, -0.08) | -2.11* | -0.64 | -1.36 |  | 0.91  (0.37, 1.44) | 3.34** | 0.06 | 0.36 | -0.04 | -0.15 | -0.24 | -1.17 |
| Never | Reference=0 | | Reference=0 | | Reference=0 | | Reference=0 | |  | Reference=0 | | Reference=0 | | Reference=0 | | Reference=0 | |
| **Avoidance of sharing utensils with other people** | | | | | | | | |  |  |  |  |  |  |  |  |  |
| Always | 0.04 | 0.22 | -0.23 | -1.58 | -0.18 | -0.99 | -0.04 | -0.22 |  | -0.37  (-0.65, -0.08) | -2.53* | -0.21  (-0.38, -0.04) | -2.45* | -0.18 | -1.37 | -0.28  (-0.50, -0.07) | -2.59* |
| Most of the time | 0.22 | 1.16 | 0.001 | 0.004 | -0.05 | -0.25 | 0.01 | 0.08 |  | 0.11 | 0.69 | -0.18 | -1.89 | -0.02 | -0.14 | -0.16 | -1.27 |
| Sometimes | 0.15 | 0.70 | -0.13 | -0.75 | -0.21 | -0.91 | 0.14 | 0.70 |  | 0.15 | 0.89 | -0.16 | -1.58 | -0.05 | -0.32 | -0.26  (-0.51, -0.003) | -1.99* |
| Occasionally | -0.06 | -0.25 | -0.12 | -0.60 | -0.29 | -1.16 | 0.11 | 0.54 |  | 0.22 | 1.16 | 0.02 | 0.19 | 0.24 | 1.39 | 0.02 | 0.15 |
| Never | Reference=0 | | Reference=0 | | Reference=0 | | Reference=0 | |  | Reference=0 | | Reference=0 | | Reference=0 | | Reference=0 | |
| **Practice of hand hygiene with soap and water** | | | | | | | | |  |  |  |  |  |  |  |  |  |
| Always | -0.55 | -1.46 | -0.83  (-1.44, -0.21) | -2.62** | -0.83  (-1.62, -0.05) | -2.08* | -0.09 | -0.26 |  | -0.10 | -0.38 | -0.53  (-0.83, -0.23) | -3.49** | -0.76  (-1.23, -0.29) | -3.17** | -0.81  (-1.20, -0.43) | -4.13*** |
| Most of the time | -0.87  (-1.64, -0.09) | -2.19* | -1.03  (-1.67, -0.39) | -3.14** | -1.12  (-1.93, -0.30) | -2.68** | -0.25 | -0.68 |  | 0.45 | 1.66 | -0.44  (-0.75, -0.14) | -2.82** | -0.57  (-1.05, -0.08) | -2.29* | -0.69  (-1.09, -0.29) | -3.41** |
| Sometimes | -0.31 | -0.59 | -0.61 | -1.43 | -0.72 | -1.33 | 0.31 | 0.66 |  | 0.22 | 0.77 | -0.54  (-0.87, -0.21) | -3.19** | -0.53  (-1.05, -0.01) | -1.99* | -0.77  (-1.19, -0.34) | -3.54*** |
| Occasionally | -1.29 | -1.87 | -1.37  (-2.50, -0.25) | -2.39* | -1.36 | -1.86 | -0.20 | -0.31 |  | 0.50 | 1.52 | -0.45  (-0.82, -0.07) | -2.32* | -0.81  (-1.40, -0.22) | -2.69** | -0.73  (-1.22, -0.25) | -2.98** |
| Never | Reference=0 | | Reference=0 | | Reference=0 | | Reference=0 | |  | Reference=0 | | Reference=0 | | Reference=0 | | Reference=0 | |
| **Practice of hand hygiene immediately after coughing and sneezing** | | | | | | | | |  |  |  |  |  |  |  |  |  |
| Always | 0.32 | 1.31 | -0.07 | -0.32 | 0.09 | 0.35 | 0.07 | 0.33 |  | -0.52  (-0.89, -0.15) | -2.74** | -0.58  (-0.80, -0.37) | -5.29*** | -0.62  (-0.96, -0.28) | -3.57*** | -0.59  (-0.87, -0.31) | -4.13*** |
| Most of the time | 0.12 | 0.49 | -0.06 | -0.30 | -0.18 | -0.69 | -0.002 | -0.01 |  | 0.05 | 0.24 | -0.44  (-0.67, -0.22) | -3.81*** | -0.39  (-0.75, -0.03) | -2.14* | -0.44  (-0.74, -0.14) | -2.92** |
| Sometimes | 0.18 | 0.73 | -0.02 | -0.11 | -0.18 | -0.69 | 0.01 | 0.06 |  | -0.03 | -0.14 | -0.45  (-0.69, -0.21) | -3.67*** | -0.47  (-0.85, -0.09) | -2.41* | -0.43  (-0.74, -0.12) | -2.72** |
| Occasionally | -0.01 | -0.03 | -0.19 | -0.85 | -0.30 | -1.08 | -0.09 | -0.38 |  | 0.07 | 0.29 | -0.54  (-0.80, -0.29) | -4.15*** | -0.53  (-0.94, -0.13) | -2.57* | -0.46  (-0.79, -0.12) | -2.69** |
| Never | Reference=0 | | Reference=0 | | Reference=0 | | Reference=0 | |  | Reference=0 | | Reference=0 | | Reference=0 | | Reference=0 | |
| **Use of a face mask** | | | | | | | | |  |  |  |  |  |  |  |  |  |
| Always | -0.09  (-0.15, -0.03) | -2.84** | -0.05  (-0.11, -0.001) | -1.99* | -0.06 | -1.79 | -0.01 | -0.37 |  | -0.36 | -0.80 | -0.95  (-1.47, -0.44) | -3.62*** | -1.01  (-1.83, -0.20) | -2.44* | -1.22  (-1.88, -0.55) | -3.59*** |
| Most of the time | -0.03 | -0.75 | -0.01 | -0.23 | -0.04 | -0.95 | 0.03 | 0.89 |  | 0.04 | 0.09 | -0.87  (-1.40, -0.34) | -3.24** | -0.82 | -1.94 | -1.01  (-1.68, -0.33) | -2.91** |
| Sometimes | -0.05 | -1.14 | 0.01 | 0.41 | 0.02 | 0.35 | 0.01 | 0.32 |  | -0.06 | -0.13 | -0.89  (-1.45, -0.33) | -3.14** | -0.88 | -1.96 | -1.09  (-1.81, -0.37) | -2.99** |
| Occasionally | -0.03 | -0.71 | 0.001 | 0.02 | 0.02 | 0.32 | 0.04 | 0.90 |  | 0.43 | 0.78 | -0.84  (-1.47, -0.21) | -2.60* | -0.65 | -1.29 | -1.01  (-1.82, -0.20) | -2.45* |
| Do not know | Reference=0 | | Reference=0 | | Reference=0 | | Reference=0 | |  | Reference=0 | | Reference=0 | | Reference=0 | | Reference=0 | |
| **The practice of hand hygiene after touching contaminated objects** | | | | | | | | |  |  |  |  |  |  |  |  |  |
| Always | 0.001 | 0.004 | -0.48 | -1.74 | -0.07 | -0.19 | 0.19 | 0.63 |  | -0.07 | -0.16 | -0.79  (-1.28, -0.30) | -3.17** | -1.04  (-1.80, -0.27) | -2.65** | -1.11  (-1.74, -0.48) | -3.48** |
| Most of the time | 0.02 | 0.06 | -0.46 | -1.59 | -0.07 | -0.19 | 0.24 | 0.76 |  | 0.33 | 0.75 | -0.74  (-1.24, -0.24) | -2.92** | -0.86  (-1.64, -0.08) | -2.16* | -0.95  (-1.59, -0.31) | -2.92** |
| Sometimes | -0.14 | -0.35 | -0.58 | -1.82 | -0.03 | -0.07 | 0.28 | 0.80 |  | 0.54 | 1.11 | -0.64  (-1.20, -0.08) | -2.26* | -0.66 | -1.48 | -0.90  (-1.62, -0.18) | -2.46* |
| Occasionally | -0.06 | -0.13 | -0.56 | -1.49 | 0.01 | 0.03 | 0.25 | 0.62 |  | 0.65 | 1.13 | -0.46 | -1.39 | -0.44 | -0.85 | -0.56 | -1.31 |
| Never | Reference=0 | | Reference=0 | | Reference=0 | | Reference=0 | |  | Reference=0 | | Reference=0 | | Reference=0 | | Reference=0 | |
| **The COVID-19 pandemic had caused unnecessary worry** | | | | | | | | |  |  |  |  |  |  |  |  |  |
| Always | 0.62 | 1.64 | 0.66  (0.04, 1.27) | 2.10* | 0.89  (0.11, 1.67) | 2.23* | 0.97  (0.30, 1.64) | 2.85** |  | -0.37  (-0.64, -0.10) | -2.66** | 0.06 | 0.73 | 0.04 | 0.29 | 0.02 | 0.20 |
| Most of the time | 0.01 | 0.06 | 0.21 | 1.36 | 0.002 | 0.01 | 0.06 | 0.32 |  | 0.10 | 0.62 | 0.19  (0.003, 0.37) | 1.99* | 0.38  (0.09, 0.67) | 2.61** | 0.28  (0.04, 0.51) | 2.31* |
| Sometimes | -0.15 | -0.90 | 0.003 | 0.02 | -0.24 | -1.36 | -0.01 | -0.08 |  | 0.02 | 0.17 | -0.09 | -1.28 | -0.08 | -0.73 | -0.08 | -0.90 |
| Occasionally | 0.10 | 0.54 | 0.13 | 0.84 | -0.08 | -0.44 | 0.06 | 0.37 |  | -0.07 | -0.49 | -0.05 | -0.59 | -0.01 | -0.06 | 0.05 | 0.53 |
| Never | Reference=0 | | Reference=0 | | Reference=0 | | Reference=0 | |  | Reference=0 | | Reference=0 | | Reference=0 | | Reference=0 | |

**P<*.05, ***P<*.01, ****P<*.001.

B: unstandardized coefficient.

**Table S5.** Univariate linear regression analysis of the association between information needs about COVID-19 and mental health parameters in Spanish and Chinese participants adjusted for age, gender, and education (N=1528).

| **Information needs** | **Spain** | | | | | | | |  | **China** | | | | | | | |
| --- | --- | --- | --- | --- | --- | --- | --- | --- | --- | --- | --- | --- | --- | --- | --- | --- | --- |
|  | IES-R | | DASS-21  Stress | | DASS-21  Anxiety | | DASS-21  Depression | |  | IES-R | | DASS-21  Stress | | DASS-21  Anxiety | | DASS-21  Depression | |
|  | *B*  (95%CI) | ***t*** | *B*  (95%CI) | ***t*** | *B*  (95%CI) | *t* | *B*  (95%CI) | *t* |  | *B*  (95%CI) | *t* | *B*  (95%CI) | *t* | *B*  (95%CI) | *t* | *B*  (95%CI) | *t* |
| **Symptoms** |  |  |  |  |  |  |  |  |  |  |  |  |  |  |  |  |  |
| **Yes** | 0.40  (0.20, 0.60) | 3.93*** | 0.20  (0.03, 0.37) | 2.34* | 0.29  (0.08, 0.51) | 2.72** | 0.16 | 1.75 |  | 0.26  (0.02, 0.51) | 2.09* | 0.08 | 1.06 | 0.20 | 1.70 | 0.07 | 0.72 |
| **No** | Reference=0 | | Reference=0 | | Reference=0 | | Reference=0 | |  | Reference=0 | | Reference=0 | | Reference=0 | | Reference=0 | |
| **Prevention methods** |  |  |  |  |  |  |  |  |  |  |  |  |  |  |  |  |  |
| **Yes** | 0.49  (0.30, 0.69) | 4.96*** | 0.32  (0.15, 0.48) | 3.80*** | 0.38  (0.17, 0.58) | 3.57*** | 0.18 | 1.92 |  | 0.30  (0.01, 0.59) | 2.00* | -0.06 | -0.64 | -0.06 | -0.46 | -0.10 | -0.86 |
| **No** | Reference=0 | | Reference=0 | | Reference=0 | | Reference=0 | |  | Reference=0 | | Reference=0 | | Reference=0 | | Reference=0 | |
| **Management methods** |  |  |  |  |  |  |  |  |  |  |  |  |  |  |  |  |  |
| **Yes** | 0.37  (0.16, 0.58) | 3.50*** | 0.19  (0.02, 0.36) | 2.15* | 0.15 | 1.33 | 0.03 | 0.33 |  | 0.23  (0.03, 0.44) | 2.24* | 0.09 | 1.55 | 0.22  (0.03, 0.41) | 2.31* | 0.09 | 1.13 |
| **No** | Reference=0 | | Reference=0 | | Reference=0 | | Reference=0 | |  | Reference=0 | | Reference=0 | | Reference=0 | | Reference=0 | |
| **Regular information update** |  |  |  |  |  |  |  |  |  |  |  |  |  |  |  |  |  |
| **Yes** | 0.12 | 1.17 | -0.03 | -0.37 | -0.01 | -0.10 | -0.08 | -0.87 |  | 0.41  (0.003, 0.81) | 1.98* | -0.13 | -1.11 | -0.11 | -0.60 | -0.18 | -1.19 |
| **No** | Reference=0 | | Reference=0 | | Reference=0 | | Reference=0 | |  | Reference=0 | | Reference=0 | | Reference=0 | | Reference=0 | |
| **More personalized information, such as those with preexisting medical conditions** | | | | | | | | |  |  |  |  |  |  |  |  |  |
| **Yes** | 0.48  (0.28, 0.68) | 4.75*** | 0.25  (0.09, 0.42) | 3.00** | 0.37  (0.16, 0.59) | 3.50** | 0.27  (0.09, 0.45) | 2.92** |  | 0.05 | 0.35 | -0.11 | -1.28 | -0.11 | -0.82 | -0.16 | -1.54 |
| **No** | Reference=0 | | Reference=0 | | Reference=0 | | Reference=0 | |  | Reference=0 | | Reference=0 | | Reference=0 | | Reference=0 | |
| **Effectiveness of drugs and vaccines** | | | | | | | | |  |  |  |  |  |  |  |  |  |
| **Yes** | 0.35  (0.12, 0.58) | 2.97** | 0.16 | 1.67 | 0.11 | 0.84 | 0.08 | 0.71 |  | 0.10 | 0.56 | -0.15 | -1.52 | -0.09 | -0.54 | -0.20 | -1.56 |
| **No** | Reference=0 | | Reference=0 | | Reference=0 | | Reference=0 | |  | Reference=0 | | Reference=0 | | Reference=0 | | Reference=0 | |
| **Number of infected by geographical location** | | | | | | | | |  |  |  |  |  |  |  |  |  |
| **Yes** | 0.11 | 1.09 | 0.06 | 0.76 | 0.11 | 1.04 | 0.02 | 0.20 |  | 0.15 | 0.89 | -0.09 | -0.89 | -0.16 | -1.05 | -0.19 | -1.52 |
| **No** | Reference=0 | | Reference=0 | | Reference=0 | | Reference=0 | |  | Reference=0 | | Reference=0 | | Reference=0 | | Reference=0 | |
| **Travel advice** |  |  |  |  |  |  |  |  |  |  |  |  |  |  |  |  |  |
| **Yes** | 0.21  (0.01, 0.41) | 2.10* | 0.15 | 1.83 | 0.09 | 0.84 | 0.03 | 0.34 |  | 0.12 | 0.90 | -0.15 | -1.84 | -0.05 | -0.41 | -0.24  (-0.44, -0.03) | -2.29* |
| **No** | Reference=0 | | Reference=0 | | Reference=0 | | Reference=0 | |  | Reference=0 | | Reference=0 | | Reference=0 | | Reference=0 | |
| **Transmission method** |  |  |  |  |  |  |  |  |  |  |  |  |  |  |  |  |  |
| **Yes** | 0.50  (0.31, 0.69) | 5.11*** | 0.31  (0.15, 0.47) | 3.82*** | 0.38  (0.17, 0.58) | 3.63*** | 0.24  (0.07, 0.42) | 2.68** |  | 0.48  (0.13, 0.83) | 2.66** | -0.10 | -0.93 | -0.14 | -0.84 | -0.13 | -0.95 |
| **No** | Reference=0 | | Reference=0 | | Reference=0 | | Reference=0 | |  | Reference=0 | | Reference=0 | | Reference=0 | | Reference=0 | |
| **Other countries’ response** |  |  |  |  |  |  |  |  |  |  |  |  |  |  |  |  |  |
| **Yes** | 0.12 | 1.21 | 0.12 | 1.41 | -0.02 | -0.20 | -0.01 | -0.11 |  | 0.23  (0.05, 0.41) | 2.47* | -0.02 | -0.32 | -0.12 | -1.44 | -0.15  (-0.29, -0.02) | -2.18* |
| **No** | Reference=0 | | Reference=0 | | Reference=0 | | Reference=0 | |  | Reference=0 | | Reference=0 | | Reference=0 | | Reference=0 | |

**P<*.05, ***P<*.01, ****P<*.001.

B: unstandardized coefficient.
